# Microbiota Composition Shaped by Seasonal Variation and Environmental Compartments Affects the Abundance of Antimicrobial‐Resistant Bacteria in Indonesian Aquaculture

**DOI:** 10.1111/1758-2229.70337

**Published:** 2026-04-14

**Authors:** Hajime Nakatani, Noor Hidhayati, Dien Arista Anggorowati, Aoi Kaji, Kaho Tobioka, Saki Ishiguro, Stephanie Angela Yosiano, Marsiti Apriastini, Khairul Anam, Dwi Susilaningsih, Koji Mitsui, Kotone Yamamoto, Sae Tanaka, Idham Sumarto Pratama, Asep Ridwanudin, Tomoko Arakawa, Fumiyoshi Okazaki

**Affiliations:** ^1^ Department of Biomolecular Engineering, Graduate School of Engineering Nagoya University Nagoya Japan; ^2^ Research Center for Applied Microbiology, Research Organization for Life Sciences and Environment National Research and Innovation Agency (BRIN) Cibinong Indonesia; ^3^ Research Center for Marine and Land Bioindustry National Research and Innovation Agency (BRIN) Pemenang Indonesia; ^4^ Department of Life Sciences, Graduate School of Bioresources Mie University Tsu Japan; ^5^ Research Center for Applied Zoology National Research and Innovation Agency (BRIN) Cibinong Indonesia; ^6^ LaLa Product Ltd. Nagoya Japan

**Keywords:** antimicrobial resistance, aquaculture, Indonesia, microbial community, tetracycline

## Abstract

Antimicrobial use in aquaculture can drive antimicrobial‐resistant (AMR) bacteria, but the ecological factors shaping their emergence and persistence across environmental compartments remain unclear. We investigated these factors through laboratory experiments and field surveys in Indonesia. In zebrafish aquaria, oxytetracycline exposure promoted the emergence of resistant bacteria and mainly affected the gut microbiota. In field surveys, gene‐based analyses identified *tetG*‐ and ribosomal protection protein‐associated taxa across sample types. Bacterial community analysis revealed seasonal contrasts: dry‐season samples frequently clustered into a “main group” enriched in AMR‐associated taxa, whereas wet‐season samples showed fewer members and greater heterogeneity. Mapping of resistant taxa indicated higher proportions of resistant bacteria in the main group and during the dry season. The median relative abundance of potential tet‐resistant bacteria was higher in the main group than in other samples (3.52% vs. 1.34%) and in the dry season than in the wet season (3.34% vs. 1.80%). Quantitative comparisons among environmental compartments suggested that the gut functions as a primary site for the emergence of AMR bacteria, while water serves as a medium for their accumulation and dispersal. These findings indicate that seasonal variation and compartment‐specific microbial structure jointly shape the abundance and distribution of AMR bacteria in aquaculture environments.

## Graphical abstract

Antibiotics reshape fish gut microbiota, promoting antimicrobial‐resistant bacteria (ARB) emergence. In the dry season, ARB accumulate in water and dominate a specific niche. Wet‐season hydrological changes introduce new bacteria, disturb native communities and drive shifts in AMB abundance and the emergence of novel resistant taxa across compartments.

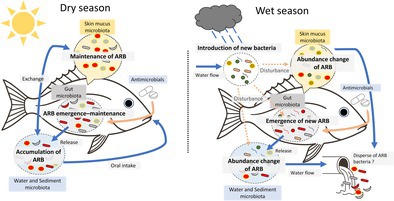

## 1 Introduction

As seafood becomes an increasingly important global source of dietary protein, developing a supply system that ensures both food safety and minimal environmental impact is essential (FAO 2024). However, achieving sustainable aquaculture requires addressing several challenges, including disease control, wastewater treatment and the development of environmentally responsible feed and farming practices (FAO 2024; Kelling et al. 2023; Troell et al. 2023). Among these, conventional disease control strategies involving antimicrobial use pose substantial risks to both human health and the environment (FAO 2024; Ma et al. 2021; Aslam et al. 2024; Desbois et al. 2025; Milijasevic et al. 2024; Pepi and Focardi 2021; Eckert et al. 2019; Cantas et al. 2013; Cherian et al. 2023). These risks are not confined to national borders, making antimicrobial resistance (AMR) a significant global concern (FAO 2024; Aslam et al. 2024; Desbois et al. 2025; Milijasevic et al. 2024; Cherian et al. 2023).

In aquaculture, antibiotics are the primary class of antimicrobials used, with an estimated application rate of 164.8 mg per kg of seafood in 2017—higher than the average rate reported for terrestrial livestock (approximately 121 mg/kg) (Schar et al. 2020). Typically delivered via medicated feed, antibiotics are released into the aquatic environment, where they become widely dispersed and are suspected to pose ecological risks (Desbois et al. 2025; Milijasevic et al. 2024; Eckert et al. 2019; Cantas et al. 2013). Several studies have reported that antibiotic‐laden effluents may disrupt natural microbial communities, impair nutrient cycling and destabilise symbiotic interactions (Eckert et al. 2019; Cantas et al. 2013). Moreover, antibiotic exposure promotes the emergence and persistence of antimicrobial‐resistant bacteria (ARB), which may infect humans through contaminated seafood or water and pose serious public health risks (Milijasevic et al. 2024; Pepi and Focardi 2021; Cherian et al. 2023; Elbashir et al. 2018; Martins et al. 2019).

Fish host diverse microbial communities on their skin and in their gut, many of which originate from the surrounding aquatic environment (Elbashir et al. 2018; Ziarati et al. 2022; Sugita et al. 1995; Sokołowska et al. 2005; Smith and Gradon 2013; Cortés‐Sánchez et al. 2021). Some of these bacteria are known pathogens or opportunistic organisms capable of causing infections in immunocompromised individuals. ARB and antibiotic resistance genes have been detected in these microbial communities (Elbashir et al. 2018; Martins et al. 2019), raising concerns about treatment failure and increased mortality, particularly among vulnerable populations (Ahmed et al. 2024). In addition, the presence of antibiotic‐resistant fish pathogens has been reported (Miller Ron and Harbottle 2018), complicating disease management and threatening the stability of aquaculture systems. Thus, understanding the mechanisms underlying the emergence and distribution of ARB in aquaculture environments is essential to assess AMR‐related risks to both humans and fish.

Southeast Asia is a major aquaculture‐producing region, cultivating freshwater species such as catfish and tilapia, and marine species including shrimp, seabass and shellfish (FAO 2024; NACA, n.d.; Needham and Funge‐Smith 2014; Luo et al. 2022). While several countries have introduced national guidelines for the responsible use of antibiotics in aquaculture (Desbois et al. 2025), enforcement and implementation remain inconsistent. As production continues to expand, concerns persist regarding the escalation of antibiotic resistance and environmental degradation (Schar et al. 2020, 2021). According to Schar et al., global antimicrobial use in aquaculture is projected to increase from approximately 10,259 t in 2017 to about 13,600 t by 2030, representing a 33% increase. Of this, 93.8% is attributed to the Asia‐Pacific region, with usage in countries such as Indonesia and Vietnam expected to rise by up to 55% by 2030 (Schar et al. 2020).

Although previous studies have shown that antibiotic use in aquaculture can promote the spread of resistant bacteria and alter microbial communities, the specific ecological mechanisms driving the emergence and distribution of ARB across fish‐associated and environmental compartments remain poorly understood. In particular, few studies have integrated experimental models with field‐based observations to assess how antibiotic pressure affects microbiota structure and resistance patterns in aquaculture ecosystems.

In this study, we combined laboratory experiments with field surveys in Indonesian aquaculture to assess how antibiotic use influences bacterial communities and the emergence of resistant bacteria. Oxytetracycline (OTC) was selected as the model antibiotic because it is widely used in aquaculture and is relevant to the emergence of tetracycline‐resistant bacteria in fish farming environments. Whereas many earlier studies focused either on controlled laboratory settings or on field surveys alone, our work integrates both approaches. This combined framework helps interpret seasonal and environmental patterns of AMR in aquaculture in light of experimentally supported changes in bacterial community structure. Specifically, we aimed to determine how antibiotic exposure, environmental compartments and seasonal variation shape the emergence, persistence and distribution of resistant bacteria in aquaculture environments.

## 2 Experimental Procedures

### 2.1 Laboratory Experiments for Antimicrobial Resistance in an Aquarium System

Adult zebrafish ( *Danio rerio* ) were obtained from a commercial supplier in Japan and acclimated in a 60 × 30 × 36 cm glass aquarium for at least 2 weeks prior to experimentation. The aquarium was maintained at 28°C, and fish were fed every 12 h using an automated feeder.

To assess the emergence of ARB in the aquarium environment, 12 zebrafish were transferred into an overflow aquarium system (CROSS MINI NWC‐341; NISSO, Osaka, Japan). OTC hydrochloride (OTC; Tokyo Chemical Industry Co. Ltd., Tokyo, Japan) was mixed into a commercial fish feed (TetraMin Super 17,653; Spectrum Brands Japan, Kanagawa, Japan) at 5 mg/g feed. The treatment group received 0.3 g of the OTC‐supplemented feed per administration, twice weekly for 4 weeks, whereas the control group received 0.3 g of the same feed without OTC supplementation on the same schedule.

Samples were collected at four time points: Day 0 (baseline), Week 1, Week 2 and Week 4 (Figure S1A). Epidermal mucus was collected using sterile swabs, and gut contents were obtained from euthanised fish. Water samples (50 mL) were collected using sterile syringes. All samples were stored at −30°C until DNA extraction.

### 2.2 Cultivation and Isolation of ARB

Samples were collected from both a laboratory aquarium system used for zebrafish ( *D. rerio* ) experiments and two aquaculture farms on Lombok Island, Indonesia, rearing tilapia ( *Oreochromis niloticus* ) and catfish ( *Clarias batrachus* ). The two farms were located in West Lombok Regency, West Nusa Tenggara Province, with Facility A in Lingsar and Facility B in Batu Kumbung. Sample types included fish epidermal mucus, gut content, pond water and sediment. The aquaculture farms operated using a flow‐through system with river water, and the primary antibiotics used were tetracycline and OTC (personal communication).

Epidermal mucus was collected by gently scraping the fish with a sterile instrument under anaesthesia. Gut contents were obtained post‐euthanasia via dissection. Water samples (500 mL) were taken from two to three sites per pond. Sediment was collected alongside water and processed through a stainless‐steel mesh.

Samples were suspended in sterile water and plated onto Nutrient Agar and R2A Agar supplemented with 50 μg/mL of OTC or tetracycline. Plates were incubated at 28°C for 2–6 days. Colonies with distinct morphology were re‐streaked for isolation.

For species identification, full‐length 16S rRNA genes were amplified using colony PCR, separated by agarose gel electrophoresis, purified and sequenced. Species were identified by BLASTn searches against the NCBI 16S rRNA gene database. Primer sequences are listed in Table S1.

### 2.3 Sample Collection, Environmental DNA Preparation and Amplicon Library Construction

Swabs used for epidermal mucus collection were stored in DNA/RNA Shield (Zymo Research, Irvine, CA, USA) and kept at 4°C until extraction. Water samples were filtered through 0.2 μm cellulose membrane filters (47 mm diameter; ADVANTEC, Tokyo, Japan), and the filters were stored in DNA/RNA Shield at 4°C. Gut contents and sediments were placed in DNA/RNA Shield immediately after collection.

Genomic DNA was extracted using the PowerSoil DNA Isolation Kit (QIAGEN, Venlo, Netherlands) according to the manufacturer's instructions. 16S rRNA gene amplicon libraries were prepared for iSeq 100 sequencing (Illumina, San Diego, CA, USA). The V1–V2 or V4 regions of the 16S rRNA gene were amplified by 30‐cycle PCR using primers with overhangs for adapter ligation (Table S1), as described previously (Nakatani and Hori 2021).

For tetracycline resistance gene analysis, efflux pump and ribosomal protection protein (RPP) genes were amplified from environmental DNA using universal primers (Table S1). Amplicons were pooled by sample type, gel‐purified and used for library construction on the MinION nanopore sequencer (Oxford Nanopore Technologies plc, Oxford, UK).

Libraries were barcoded using the Rapid Barcoding Kit 24 V14 (SQK‐RBK114.24) and loaded onto R10.4.1 flow cells (FLO‐MIN114) according to the manufacturer's instructions. Base‐calling was performed in real time using MinKNOW (Windows 11), and FASTQ files were generated. Reads < 200 bp or with Q‐scores < 9 were excluded.

### 2.4 In Silico Analysis of 16S rRNA Gene Sequences

Sequencing data were analysed using CLC Genomics Workbench with the Microbial Genomics Module (QIAGEN). Paired‐end FASTQ files from the iSeq 100 platform were merged and quality‐trimmed (250–310 bp). If merging was unsuccessful, R1 reads (150 bp) were used. Metadata (sampling season, day, site, fish species, OTC treatment) were compiled.

Operational taxonomic units (OTUs) were clustered using the SILVA 16S rRNA database v132 (97% similarity; https://www.arb‐silva.de; accessed 16 October 2021). Unassigned reads were reclustered at 94% similarity. OTU tables were exported to Excel (Supporting Information: Sheets 1 and 2).

For V4 region data, paired‐end R1 and R2 reads were merged, and only merged reads with a length of approximately 300 bp were retained for downstream analysis. For V1–V2 region data, only R1 reads (~150 bp) were used due to low merging efficiency. Representative sequences of each OTU were subjected to BLASTn searches against the NCBI 16S rRNA gene database to assign taxonomic identities.

Beta diversity was assessed using unweighted UniFrac distance (Lozupone et al. 2011), followed by 3D principal coordinate analysis (PCoA) (Nakatani and Hori 2021). Differential OTU abundance analysis was conducted between OTC‐treated and control groups within each beta‐diversity‐defined cluster. OTUs with mean counts > 100 were visualised using volcano plots (log_2_ fold‐change vs. −log_10_ FDR‐adjusted *p*).

### 2.5 Analysis of Tetracycline Resistance Genes and Potential ARB

Nanopore FASTQ files were merged and trimmed (200–1500 bp). For each site, 100,000 reads were randomly sampled and mapped to antimicrobial resistance gene reference sequences (amr_target.fa; NCBI NDARO, accessed 2024‐12‐19).

Mapped reads were counted per gene, and relative abundances were visualised as heatmaps. Consensus sequences were generated and BLASTn‐searched against NCBI type strains (≥ 95% identity). Associated bacterial species were recorded (Supporting Information: Sheet 2).

Identified species' 16S rRNA sequences were retrieved and used for additional mapping. Reads were also mapped to 16S rRNA genes of tetracycline‐resistant isolates and predicted ARB (Nakatani et al. 2022).

Rarefaction analysis of phylogenetic diversity (Miller et al. 2018) was conducted using subsampling (1–20,000 reads), and based on the resulting saturation curves, read counts were normalised to 3500 for laboratory samples and 5000 for field samples.

To estimate the relative abundances of predicted or isolated AMR‐associated bacteria, normalised 16S rRNA gene sequences (V4 region) were mapped to reference 16S rRNA gene sequences of candidate ARB using CLC Genomics Workbench (QIAGEN). Mapping was conducted with the following parameters: length fraction = 0.5 and similarity = 0.99, ensuring that only high‐confidence matches—those covering at least 50% of the read length with ≥ 99% sequence identity—were retained. These stringent criteria minimised false‐positive assignments and enhanced the taxonomic accuracy of read mapping. The relative abundances of each predicted or cultured AMR‐associated taxon across different sample compartments were visualised as a heatmap.

## 3 Results

### 3.1 Emergence of ARB in the Aquarium Environment Following Antibiotic Administration

To investigate the emergence of antimicrobial‐resistant (AMR) bacteria in aquaculture environments following antibiotic administration, we conducted laboratory experiments using zebrafish ( *D. rerio* ). OTC was administered by incorporating it into fish feed, allowing exposure through ingestion and subsequent diffusion into the surrounding water.

Twelve zebrafish were housed in a 10‐L aquarium and fed OTC‐supplemented feed twice weekly for 4 weeks (a total of eight administrations; Figure S1A). OTC‐resistant bacteria were isolated from epidermal mucus, gut contents and aquarium water during the first and second weeks of the experiment (Figure S1A). Samples were collected both immediately after the transfer of fish to the tank and after each OTC treatment.

Resistant bacterial colonies were detected in both OTC‐treated and control groups, although colony abundance varied by sample type and individual fish (Figure S1B). Notably, the morphology and size of OTC‐resistant colonies differed between treated and untreated groups. These findings suggest that OTC administration promoted the emergence of OTC‐resistant bacteria in the aquarium environment.

To identify the resistant bacteria, partial 16S rRNA gene sequences were amplified using colony PCR and subjected to sequencing. The resulting sequences revealed the presence of bacterial genera commonly found in aquatic environments and associated with fish, including *Aeromonas*, *Bacillus*, *Flectobacillus*, *Pseudomonas* and *Shewanella* (Table 1) (Sugita et al. 1995; Nakatani and Hori 2021; Nakatani et al. 2022; Hwang and Cho 2006; Sheu et al. 2009; Takeuchi et al. 2021; Liu et al. 2017).

**TABLE 1:** OTC resistant bacteria isolated from zebrafish 1 and 2 week‐after OTC administration.

No.	Top‐hit taxon	Samples	Identity (%)
1	*Aeromonas veronii* bv. *veronii* ATCC 35624	GC	99.76
2	*Bacillus tropicus* MCCC 1A01406	GC	99.9
3	*Flectobacillus roseus* JC289	W	99.45
4	*Pseudomonas alcaligenes* ATCC 14909	GC	99.72
5	*Pseudomonas fluvialis* ASS‐1	W	97.81
6	*Pseudomonas multiresinivorans* ATCC 700690	GC	99.79
7	*Shewanella putrefaciens* Hammer 95	EM	99.01
8	*Pseudomonas fluvialis* ASS‐1	GC	98.06
9	*P. fluvialis* ASS‐1	W	97.06
10	*Shewanella putrefaciens* Hammer 95	GC	98.67
11	*Shewanella xiamenensis* strain S4	EM	99.25

*Note:* Blue cells: OTC+, White cells: OTC‐.

Abbreviations: EM, epidermal mucus; GC, gut content; W, water.

### 3.2 Effects of OTC Administration on the Bacterial Flora in Different Parts of the Aquarium Environment

**FIGURE 1:**
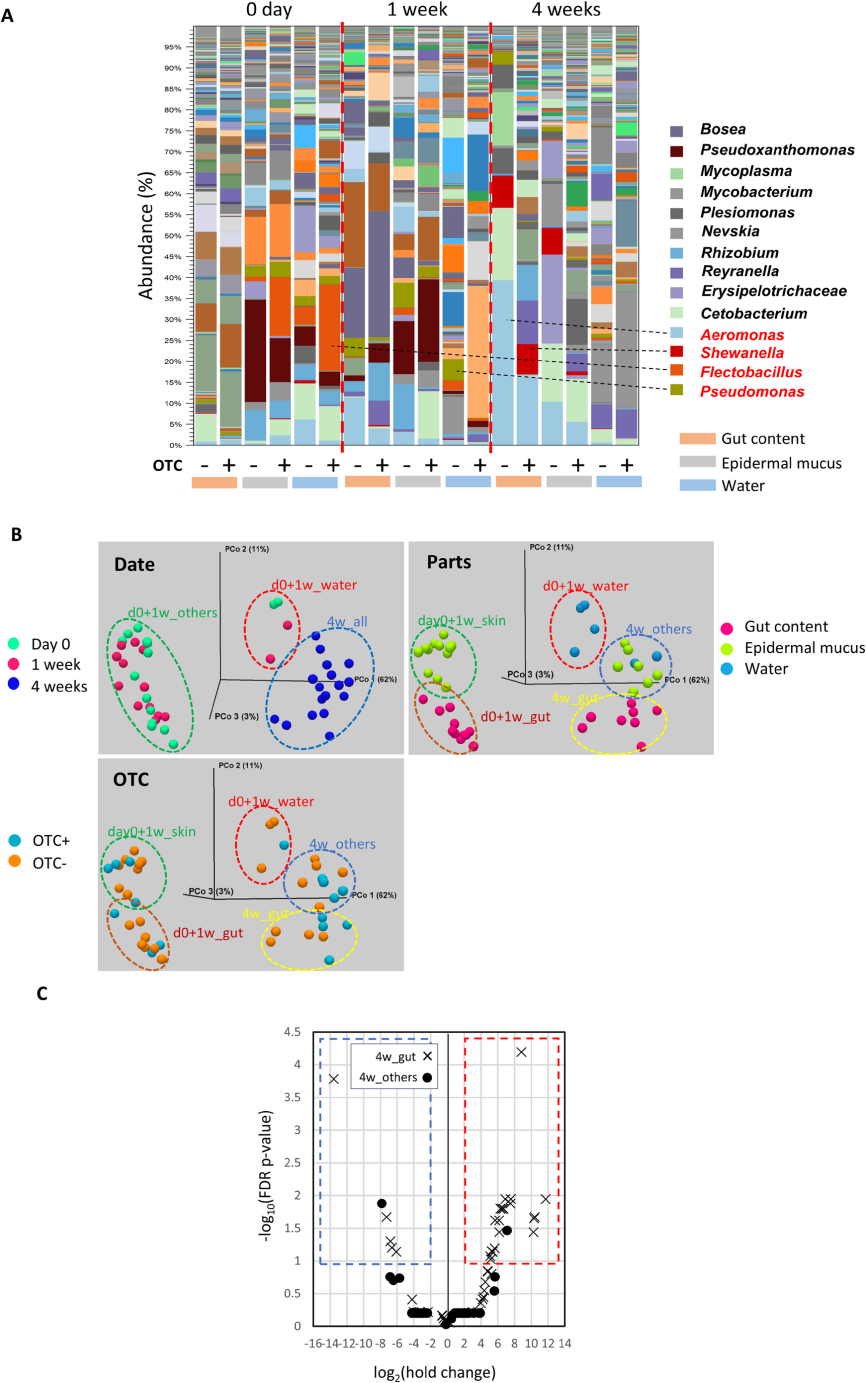
Effects of OTC administration on bacterial communities in zebrafish aquaria. (A) Bacterial communities in water, epidermal mucus and gut contents were analysed after feeding fish with OTC‐supplemented diet. Samples were also collected from a control aquarium without OTC administration. Genera corresponding to OTC‐resistant isolates identified by culture are highlighted in red. Data from replicate samples were combined and shown as stacked bar charts. (B) Beta diversity analysis using unweighted UniFrac distances was conducted to classify samples collected at Day 0, Week 1 and Week 4 from water, mucus and gut compartments. Groups of samples are indicated by dotted circles in different colours. (C) At Week 4, OTU abundances in OTC‐treated versus control groups were compared for water/mucus (4w_others ●) and gut (4w_gut ×) samples. Results are displayed in a volcano plot, showing log2 fold change relative to controls and –log10 FDR‐adjusted *p* values. OTUs with ≥ 4‐fold increases (red box) or decreases (blue box) and FDR ≤ 0.1 are highlighted.

To evaluate the effects of OTC administration on bacterial communities in the aquarium environment, we analysed the microbiota of gut contents, epidermal mucus and rearing water at day 0 (prior to treatment) and during the first and fourth weeks of the experiment. The bacterial communities were dominated by fish‐associated genera, including *Aeromonas, Shewanella, Cetobacterium, Mycoplasma and Plesiomonas* (Sugita et al. 1995; Liang et al. 2024; Zhang et al. 2022; Rasmussen et al. 2023), together with environmental genera such as *Reyranella, Bosea, Flectobacillus* (Hwang and Cho 2006; Sheu et al. 2009; Takeuchi et al. 2021; Lowrey et al. 2015; Kong et al. 2024; Gallet et al. 2023; Mondal et al. 2022) and *Pseudoxanthomonas* (Thierry et al. 2004) (Figure 1A). Genera related to cultured OTC‐resistant isolates (Table 1) were detected in all samples, regardless of OTC administration (Figure 1A). Although no marked differences in relative abundance of bacterial genera were observed at Week 1, changes appeared by Week 4, particularly in the gut and mucus samples of OTC‐treated fish compared with untreated controls (Figure 1A).

To explore the effects of OTC on bacterial communities, we first classified the samples based on their OTU compositions, since differences in bacterial community membership and taxonomic composition might influence the emergence of ARB. Beta diversity analysis using unweighted UniFrac distances (Lozupone et al. 2011)—which capture differences in community membership while accounting for phylogenetic relationships—revealed that bacterial communities clustered into two major groups according to sampling time points (Figure 1B, ‘date’, d0 + 1w_others and 4w_all). These clusters were further subdivided by sampling compartments, including water, epidermal mucus and gut content (Figure 1B, ‘parts’, d0 + 1w_skin, d0 + 1w_gut, 4w_others and 4w_gut). However, clustering was not driven by OTC administration (Figure 1B, ‘OTC’), suggesting that the antibiotic treatment did not alter the overall membership of the bacterial communities but affected the relative abundances of specific taxa (Figure 1A).

To examine which bacterial community was most affected by OTC, we analysed differentially abundant OTUs in samples from Week 4, when changes were most apparent (Figure 1A). The volcano plot (Figure 1C) indicated that most of the differentially abundant OTUs were found in the group of gut samples (4w_gut). OTUs showing ≥ 4‐fold changes with FDR‐adjusted *p* values ≤ 0.1 (Figure 1C, dotted area) are listed in Table S2. Only two OTUs in the 4w_others group (water and mucus) showed significant changes. OTUs that changed significantly in the 4w_gut group included anaerobic bacteria such as *Clostridium*, which could not be cultivated under our aerobic conditions, environmental taxa such as *Reyranella* and *Bosea*, and OUT relating to isolated OTC‐resistant bacteria such as *Shewanella putrefaciens* and *Bacillus tropicus*, which tended to increase (Table 1). OTUs that decreased included *Pseudomonas oditidis* and taxa related to *Mycoplasma*. Some OTC‐resistant isolates‐related OTUs—such as *Aeromonas veronii* and *Shewanella xiamenensis*—could not be captured by the Week 4 comparison.

These results showed that OTC administration through feed primarily influenced the gut microbiota in the aquarium environment, with little effect on the bacterial communities in water or epidermal mucus. Examination of OTU relative abundance changes at Week 4 also suggests that single time‐point, control‐based comparisons of OTU abundance are insufficient to detect the resistance‐associated bacterial species.

### 3.3 Detection of the OTC‐Resistant Related Bacteria in the Bacterial Communities in Aquarium

**FIGURE 2:**
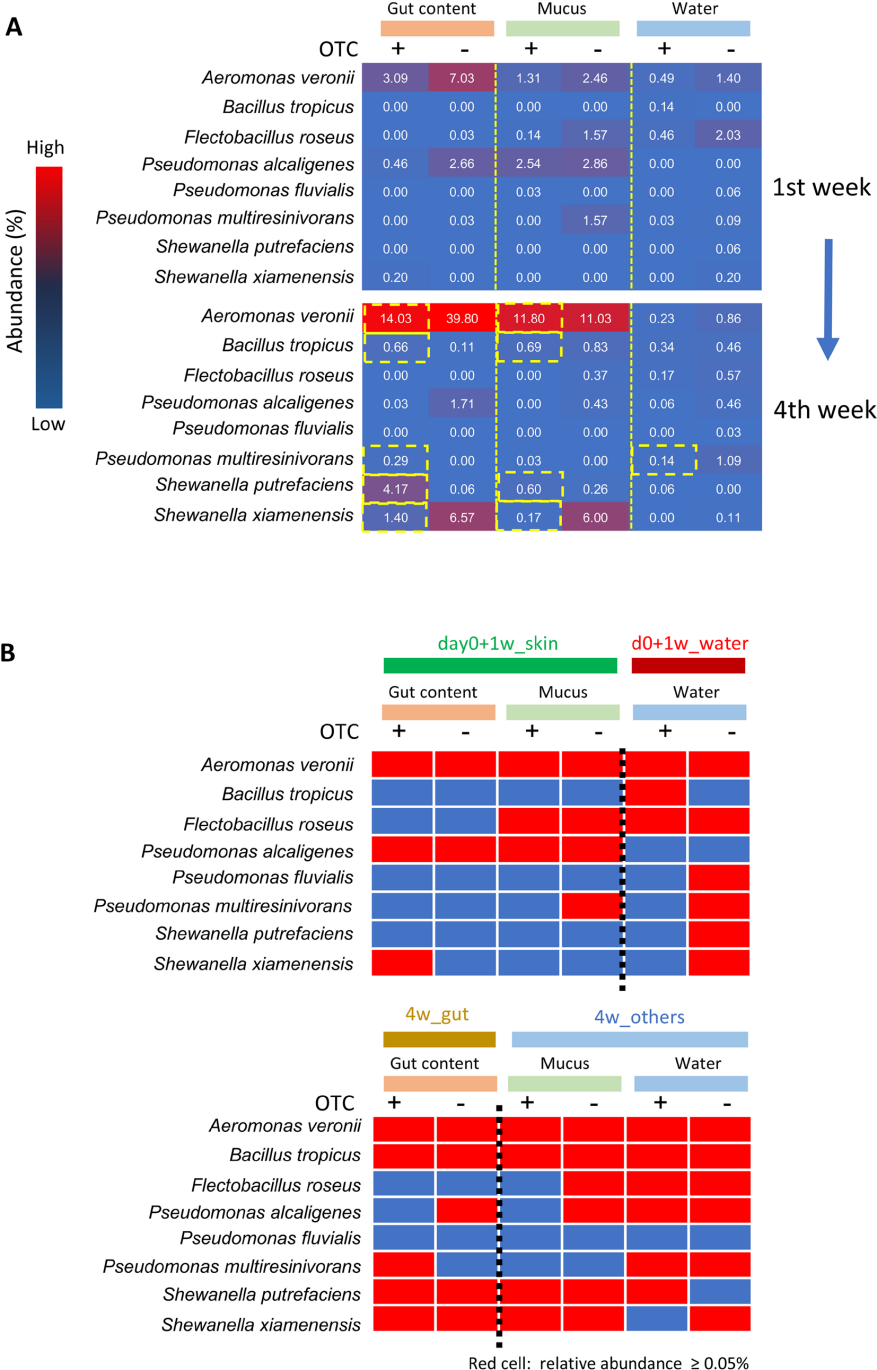
Detection of OTC‐resistant bacteria‐associated 16S rRNA genes in the bacterial flora. (A) The proportion of 16S rRNA gene sequences associated with OTC‐resistant bacteria identified through culture experiments was analysed in the sequencing data of 16S rRNA gene amplicon library obtained from each sample. Data in same condition were merged and expressed by heat map. The bacteria increased abundance by OTC administration in four weeks were highlighted by dotted‐yellow squares. (B) Detection patterns of resistance‐associated taxa across samples. Red cells indicate proportions ≥ 0.05%, and blue cells indicate < 0.05%. Samples from Week 1 and Week 4 are shown together with the community classifications defined in (B).

To further examine the dynamics of OTC‐resistant related taxa, we assessed the relative abundance of 16S rRNA gene sequences corresponding to the resistant isolates in the read data obtained by NGS. Reads affiliated with *A. veronii, Pseudomonas alcaligenes, Pseudomonas multiresinivorans* and *Flectobacillus roseus* were detected across all environmental compartments at Week 1 (Figure 2A). Their relative abundance was lower in the OTC‐treated group compared with the untreated group, suggesting antibiotic‐induced selective pressure. By Week 4, however, reads related to *A. veronii, B. tropicus, P. multiresinivorans, S. putrefaciens* and *S. xiamenensis* had increased in the gut and mucus of the OTC‐treated fish compared with Week 1, suggesting selection or enrichment under repeated OTC exposure. In contrast, reads affiliated with *F. roseus, P. alcaligenes* and *Pseudomonas fluvialis* declined in both groups by Week 4, indicating poor adaptation to the aquarium conditions. These results suggest that, in the relatively stable laboratory aquarium environment, resistant bacterial taxa became selectively enriched in specific compartments, particularly in the gut and mucus.

In untreated fish, the relative abundance of *A. veronii* and *S. xiamenensis* continued to increase throughout the experimental period (Figure 2A), remaining higher than in OTC‐treated fish. This observation indicates that single time‐point comparisons of bacterial abundance between treatment and control groups (Figure 1C) are, in some cases, insufficient to identify resistance‐associated taxa. Furthermore, among the bacterial communities shown in Figure 1B, samples belonging to the same categories exhibited similar patterns in the types of potential resistant bacteria detected at relative abundances of ≥ 0.05% (Figure 2B). This finding suggests that the community membership of bacterial flora influences the occurrence patterns of resistant taxa.

### 3.4 Isolation of Tetracycline‐Resistant Bacteria in Indonesian Aquaculture Facilities

The laboratory experiments indicated that while community profiling is useful for identifying which environmental compartments are most affected by antibiotic exposure, the reliable detection of resistant bacteria requires complementary approaches such as culture‐based isolation, sequence analysis of isolates and read mapping. It was also shown that resistance‐associated bacterial taxa could increase in relative abundance within specific environmental compartments, and that there is a relationship between the occurrence patterns of resistant taxa and the community membership of bacterial flora.

Guided by these findings, we conducted a field survey at aquaculture farms in Indonesia, where antibiotic use has raised concerns regarding the emergence of resistant bacteria (Schar et al. 2020). Samples were collected from two facilities (Facility A and Facility B) rearing catfish and Nile tilapia on Lombok Island. Targeted compartments included fish epidermal mucus, gut contents, rearing water and sediment. To assess seasonal variation in resistant bacteria, sampling was performed during both wet and dry seasons.

According to personal communication with local aquaculture farmers, antimicrobial agents containing tetracycline and OTC were used at Facility A, whereas no antibiotics were applied at Facility B. Isolation experiments yielded 70 tetracycline‐resistant (tet‐resistant) colonies from all sample types across both facilities, with a higher number of isolates obtained from Facility A (Table S3). Sequencing of the 16S rRNA gene revealed several isolates corresponding to known fish pathogens, such as *Stenotrophomonas maltophilia* , *Chryseobacterium cucumeris* (Abraham et al. 2016; Mwanza et al. 2022) and *Citrobacter freundii* (Bandeira Junior et al. 2018; Liu et al. 2024), as well as fish‐associated bacteria including *Curbibacter lanceolatus*, *Morganella morganii* and *Plesiomonas shigelloides* (Cortés‐Sánchez et al. 2021; Rodtong et al. 2005; Ke et al. 2022) (Table 2).

**TABLE 2:** Identification and characterisation of bacteria with tetracycline resistance isolated from tilapia and catfish ponds.

Sample	Facility	Top‐hit taxon	Strains	Similarity (%)	References for characteristics
Water	A	*Curvibacter lanceolatus*	ATCC 14669	98.03	—
Water	A	*Rivihabitans pingtungensis*	Npb‐03	99.39	—
Water	A	*Pseudomonas alloputida*	Kh7	100.00	—
Water	A	*Vogesella urethralis*	YM‐1	99.47	53
Sediment	A	*Acinetobacter haemolyticus*	CIP 64.3	98.77	55
Sediment	A	*Acinetobacter baumannii*	ATCC 19606	98.31	48
Sediment	A	*Serratia marcescens*	ATCC 13880	97.35	49
Sediment	A	*Shigella flexneri*	ATCC 29903	100.00	17
Mucus	A	*Chryseobacterium lecithinasegens*	PAGU 2197	99.22	—
Mucus	A	*Comamonas testosterone*	ATCC 11996	100.00	54
Mucus	A	*Serratia marcescens*	ATCC 13880	100.00	49
Mucus	A	*Vogesella urethralis*	YM‐1	99.49	53
Sediment	B	*Plesiomonas shigelloides*	NCTC 10360	99.41	19
Sediment	B	*Shigella flexneri*	ATCC 29903	100.00	17
Water	A	Chryseobacterium cucumeris*	GSE06	99.87	43
Water	A	*Citrobacter freundii* *	DSM 30039	99.87	44, 45, 56
Water	A	* Klebsiella pneumoniae subsp. Ozaenae*	ATCC 11296	99.61	50, 51
Water	A	*Massilia aerilata*	5516S‐11	99.66	—
Water	A	* Morganella morganii subsp. Morganii*	ATCC 25830	99.09	46, 57
Water	A	*Providencia alcalifaciens*	DSM 30120	99.87	58
Water	A	*Rivihabitans pingtungensis*	Npb‐03	99.51	—
Sediment	A	*Acinetobacter gyllenbergii*	CIP 110306	100.00	—
Sediment	A	*Acinetobacter modestus*	NIPH 236	99.61	—
Sediment	A	* Morganella morganii subsp. Sibonii*	DSM 14850	99.33	46, 57
Sediment	A	*Shigella flexneri*	ATCC 29903	99.73	17
Sediment	A	*Vogesella urethralis*	YM‐1	99.47	53
Mucus	A	*Acinetobacter tibetensis*	Y‐23	99.03	—
Mucus	A	*Chryseobacterium ureilyticum*	DSM 18017	99.18	—
Mucus	A	*Undibacterium squillarum*	CMJ‐15	99.83	—
Gut	A	* Morganella morganii subsp. Morganii*	ATCC 25830	99.87	46, 57
Gut	A	* Morganella morganii subsp. Sibonii*	DSM 14850	99.22	46, 57
Mucus	B	*Chryseobacterium hispalense*	DSM 25574	98.71	—
Mucus	B	*Pseudoduganella violacea*	CAVIO	98.89	—
Mucus	B	*Paucimonas lemoignei*	LMG 2207	94.49	—
Mucus	B	*Stenotrophomonas maltophilia* *	MTCC 434	98.94	42, 52

*Note:* Yellow cells: Isolates from tilapia pond. Grey cells: Isolates from catfish pond. Red text: Human Pathogens. Green text: Opportunistic human pathogens. Blue text: Opportunistic human pathogens (suspected). Asterisks: Fish pathogen (suspected).

In addition, multiple isolates were identified as potential human opportunistic pathogens, including *Acinetobacter baumannii* , *Acinetobacter haemolyticus* , *C. freundii* , *Klebsiella pneumoniae* , *Serratia marcescens* , *Comamonas testosteroni* , *Vogesella urethralis* and *S. maltophilia* (Howard et al. 2012; Zivkovic Zaric et al. 2023; Bengoechea and Sa Pessoa 2019; Paczosa Michelle and Mecsas 2016; Brooke Joanna 2012; Matsuda et al. 2023; Ryan et al. 2022; Sheck et al. 2023; Joaquin et al. 2008), as well as foodborne pathogens such as *Shigella flexneri* , *P. shigelloides* , *Providencia alcalifaciens* and *M. morganii* (Sokołowska et al. 2005; Cortés‐Sánchez et al. 2021; Rodtong et al. 2005; Kim et al. 2003; Klein et al. 2024).

To examine the genetic basis of resistance, tet‐resistance genes associated with these isolates were investigated using the Comprehensive Antibiotic Resistance Database (CARD; https://card.mcmaster.ca/home, accessed 19 December 2024). The following associations were inferred: *tetA* in *C. freundii* , *K. pneumoniae* , *M. morganii* , *P. shigelloides* , *P. alcalifaciens* , *S. marcescens* and *S. flexneri* ; *tetB* in *A. baumannii* , *C. freundii* , *M. morganii* , *P. alcalifaciens* , *S. marcescens* and *S. flexneri* ; *tetC* in *A. baumannii* and *C. testosteroni* and *tetD* in *C. freundii* , *C. testosteroni* , *K. pneumoniae* , *M. morganii* and *S. marcescens* .

### 3.5 Detection of Tet‐Resistance Gene and the Prediction of the Bacterial Species Harbouring Tet‐Resistance in Indonesian Aquaculture Environments

To complement the isolation of tet‐resistant bacteria, which identified only culturable species, we conducted a molecular survey to detect tet‐resistance genes and infer their potential bacterial hosts in Indonesian aquaculture environments. PCR was used to screen for tetracycline efflux pump genes (*tetA, B, C, D, E, G, H, J, K, L, Y*) and RPP genes (*tetBP, M, O, Q, S, T, W, otrA*).

**FIGURE 3:**
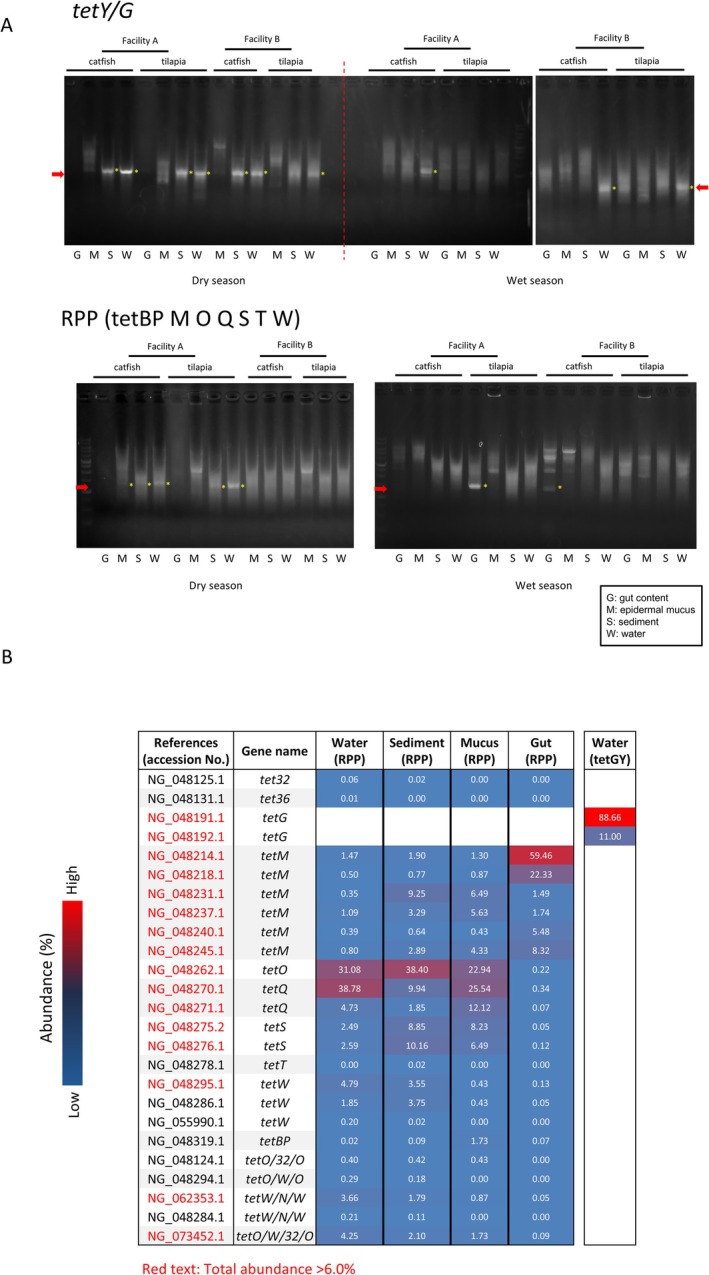
Detection of tetracycline resistance genes from environmental DNA in Indonesian aquaculture. (A) Detection of efflux pump genes and ribosomal protection protein (RPP) genes using universal primers via PCR. Efflux pump genes *tetY/G* and RPP genes were detected from some environmental DNA samples (marked with yellow *). (B) Amplified DNA from aquaculture environmental DNA via PCR (marked with yellow *) were comprehensively sequenced using a nanopore sequencer and compared to known antibiotic resistance‐related gene sequences in NCBI nucleotide database. The proportion of antibiotic resistance‐related genes detected in each sample is shown as a heatmap. Antibiotic resistance genes with a total detection frequency of 6.0% or higher are highlighted in red.

Efflux pump‐related genes were tested with multiple primer sets. Amplification was obtained most consistently with *tetG/Y*‐specific primers, particularly in water samples from both seasons and in dry‐season sediment (Figure 3A). However, the amplification signals were unclear in mucus and gut content samples. For other efflux pump genes (*tetA, B, C, D, E, H, J, K, L*), amplification was often nonspecific, making it difficult to reliably interpret the presence or absence of these genes across environmental compartments (Figure S2). Therefore, the differences in amplification patterns should be interpreted with caution, and only the tetG/Y detections were treated as relatively robust in this study. For RPP genes, PCR with universal primers (excluding *otrA*) yielded amplification from water, sediment and mucus samples collected during the dry season, and from gut content during the wet season (Figure 3A). Amplification from mucus and sediment was inconsistent. Follow‐up PCR with specific primers confirmed the presence of *tetBP*, *tetM* and *tetQ* in all universal primer‐positive samples, while *tetO*, *tetS* and *tetW* were predominantly detected in water and sediment (Figure S3A). These genes were more frequently found in dry‐season samples and at Facility A. No amplification was observed with *otrA*‐specific primers (Figure S3B).

Amplicons (~1.2 kbp for RPP genes and ~1 kbp for *tetG/Y*) were sequenced on a nanopore platform. Between 0.24% and 19.0% of reads were identified as tet‐resistance genes. Sequencing confirmed the presence of RPP‐like sequences homologous to known genes (Figure 3B). Water, sediment and mucus amplicons contained sequences similar to *tetO*, *tetQ* and *tetS*, while gut content amplicons predominantly resembled *tetM*. Other RPP genes were detected at lower relative abundances. For efflux pump genes, only *tetG* was identified, primarily in water‐derived amplicons (Figure 3B).

**FIGURE 4:**
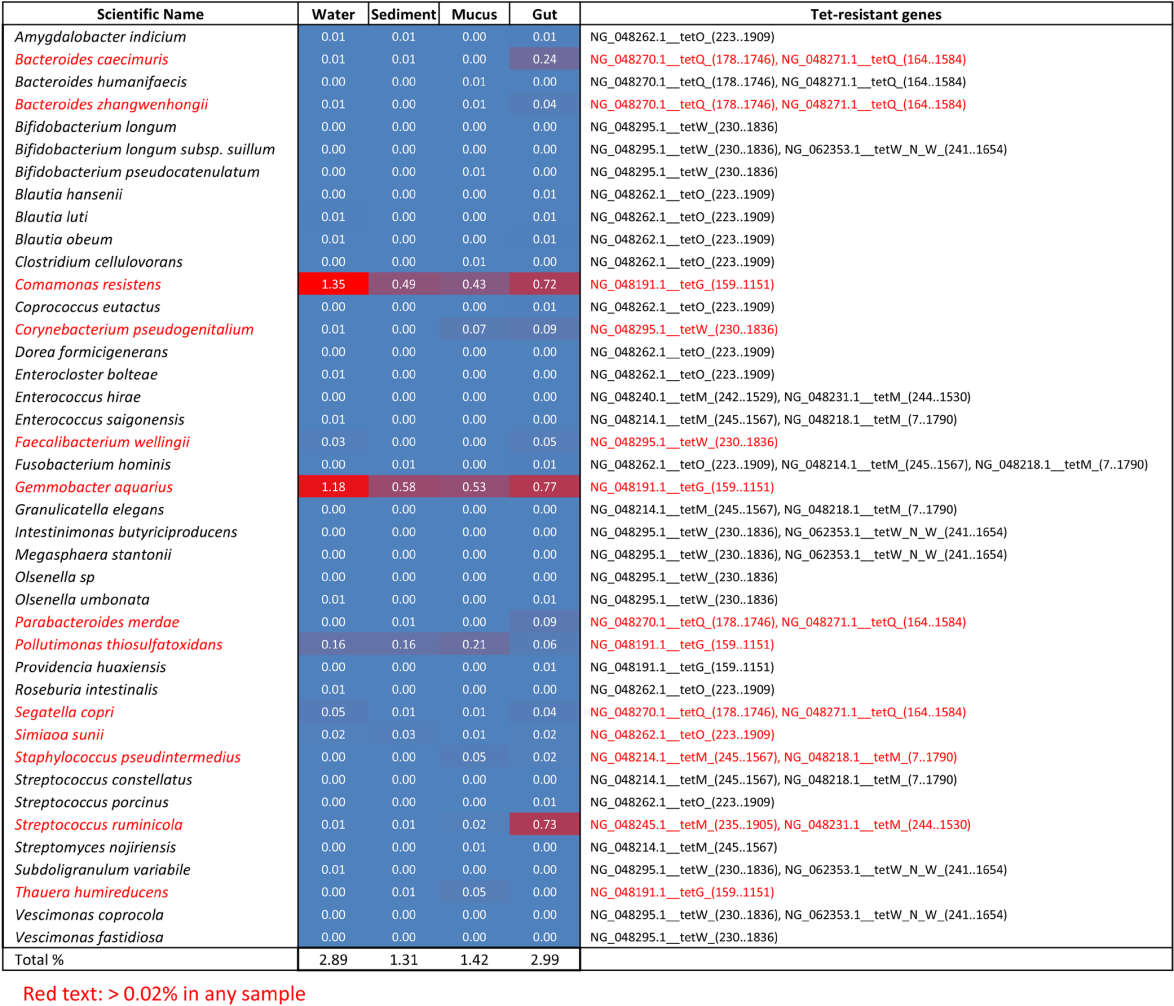
Estimation of bacterial species associated with tet‐resistance genes detected from aquaculture environments. The tetracycline resistance genes sequences identified by comprehensive sequence analysis in Figure 3 were mapped to known tetracycline resistance gene sequences, and the consensus sequences corded in the obtained sequences data were determined. Using these consensus sequences, BLASTn searches were performed against the type sequence materials in the core_n database to identify bacterial species with genes showing ≥ 95% sequence similarity to these consensus sequences. The 16S rRNA gene sequences of these identified bacterial species were retrieved and used as references to analyse the proportion of 16S rRNA gene sequences associated with these bacterial species within the 16S rRNA gene sequences obtained from environmental DNA. Bacterial species with at least one detected read were listed, and their proportions in the bacterial flora were visualised in a heatmap, linking them to their associated tet‐resistance genes. Bacterial species with detection frequencies of ≥ 0.02% in any sample are highlighted in red.

To predict potential bacterial hosts, BLASTn searches were conducted against the NCBI nucleotide database using consensus sequences from the resistance gene amplicons, restricted to type material (Figure 4). Species with ≥ 95% sequence identity were listed (Supporting Information: Sheet 2). No species‐level matches were identified for *tetS*. Next, 16S rRNA gene sequences of the matched or related species were retrieved and compared to the 16S rRNA gene sequences obtained from environmental 16S libraries to estimate their relative abundances (Figure 4).

Bacterial species associated with *tetG*, including *Comamonas resistens*, *G. aquarius* and *Pollutimonas thiosulfatoxidans*, were consistently detected across samples, with higher prevalence in water. RPP gene‐associated species were mainly found in gut contents, with lower prevalence in mucus. These included *Bacteroides caecimuris*, *Bacteroides zhangwenhongii*, *Corynebacterium pseudogenitalium*, *Faecalibacterium wellingii*, *Parabacteroides merdae* , *Segatella copri*, *Simiaoa sunii*, *Staphylococcus pseudintermedius* and *Streptococcus ruminicola* (Figure 4). Notably, *C. pseudogenitalium* and *S. pseudintermedius* are known human pathogens (Vedel et al. 2006; Maali et al. 2018).

### 3.6 Bacterial Community Analysis of Indonesian Aquaculture Environments

**FIGURE 5:**
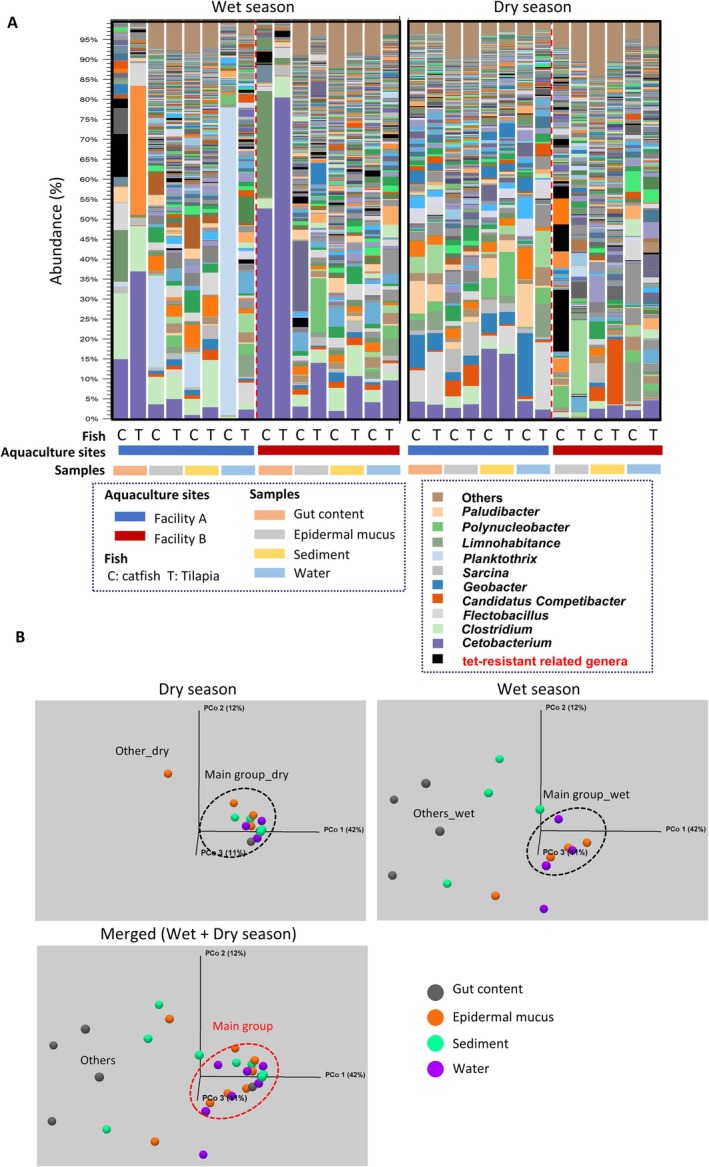
Bacterial flora analysis in Indonesian aquaculture environments. (A) The bacterial flora of different sampling sites in aquaculture environments was analysed according to sampling site, fish species, facility and sampling season. The proportion of bacterial genera present in each site is displayed as a stacked bar chart. The data represent the combined analysis results of multiple samples collected from the same sampling site, fish species, facility and sampling season. Bacterial genera associated with Tet‐resistant bacteria identified in culture experiments are indicated in black colour. (B) Beta‐diversity analysis of the bacterial flora was performed using unweighted UniFrac distance. A consolidated group (main group) formed by bacterial flora during the dry and rainy seasons is indicated with a dashed circle.

To identify potential antibiotic‐resistant bacteria within the microbial communities of aquaculture environments, we first performed a comprehensive bacterial community analysis of samples collected from Indonesian aquaculture facilities. The taxonomic composition and structure of these communities were determined (Supporting Information: Sheets 1 and 2) and visualised as stacked bar charts showing the relative abundances of bacterial genera across different sample types (Figure 5A).

The bacterial genera identified were mainly intestinal bacteria such as *Cetobacterium*, *Clostridium* and *Paludibacter* (Liang et al. 2024; Wu et al. 2012; do Vale Pereira et al. 2024), as well as genera commonly found in aquatic environments including *Flectobacillus*, *Planktothrix*, *Polynucleobacter* and *Limnohabitans* (Hwang and Cho 2006; Sheu et al. 2009; Komárek 2003; Kasalický et al. 2013; Hahn 2003) (Figure 5A). We also examined the proportions of bacterial genera potentially associated with tet‐resistant bacteria, as identified in previous culture‐based and gene‐based analyses. In most samples, these genera accounted for less than 10% of the total community. Exceptions included two samples: catfish gut content from Facility A during the wet season and catfish epidermal mucus from Facility B during the dry season (Figure 5A).

Additionally, we analysed the relative abundance of tet‐resistance associated genera in each sampling compartment. Among the compartments, gut content and epidermal mucus tended to show a relatively higher proportion of genera potentially associated with tet‐resistant bacteria compared to water or sediment samples (Figure S4).

To further explore patterns in microbial community structure, we conducted β‐diversity analysis using unweighted UniFrac distances (Lozupone et al. 2011). The results showed that during the dry season, most samples clustered into a single group, referred to as the ‘main group’ (Figure 5B, main group_dry). In contrast, wet‐season samples displayed greater variation and formed no clear cluster, except for a few samples (Figure 5B, main group_wet). Notably, those wet‐season samples that did form a group overlapped with the dry‐season main group (Figure 5B, merged: main group). This finding indicates that seasonal changes, such as changes in hydrodynamic conditions, in the aquatic environment during the wet season altered the community membership of bacterial flora across different environmental compartments.

### 3.7 Exploration of Potential Bacteria Associated With Tet‐Resistance in Indonesian Aquaculture Facilities

To assess the presence and abundance of potential tetracycline‐resistant (tet‐resistant) bacteria in aquaculture microbial communities, we examined both cultured isolates (Table 2) and additional bacterial species identified through tet‐resistance gene analysis (Figure 4, red labels). The relative abundances of 16S rRNA gene sequences closely matching these species were estimated by read mapping using their reference sequences.

**FIGURE 6:**
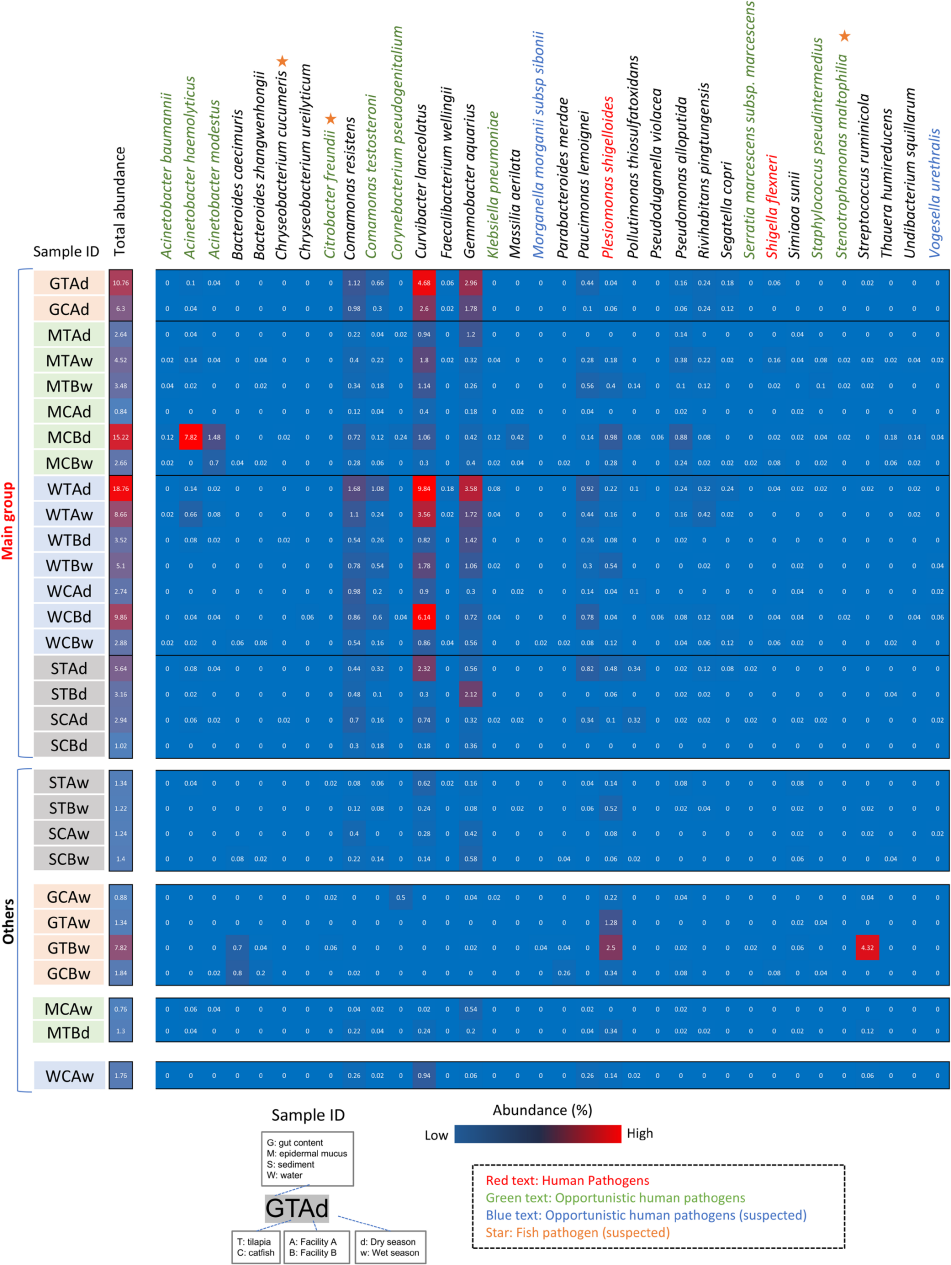
Exploration of potential tet‐resistant bacteria in the bacterial flora of Indonesian aquaculture environments. The potential tet‐resistant bacteria determined through the culture experiment (Table 2) and the analysis of tet‐resistance genes (Figure 4) were analysed to determine the proportion of 16S rRNA gene sequences associated with these bacteria within the bacterial flora of each group categorised in Figure 5B. The proportions were visualised as a heatmap for each sample. Sequence data from samples with the same sampling site, fish species, facility and season were merged and used for the analysis. Only the species detected in any sample were shown.

Several tet‐resistant species isolated from Facility A were also detected in Facility B, and vice versa (Figure 6). Samples classified within the ‘main group’ commonly contained *tetG*‐associated bacteria such as *C. resistens* and *G. aquarius*, together with cultured tet‐resistant species including *C. testosteroni* , *C. lanceolatus* and *Paucimonas lemoignei* (Figure 6). Other tet‐resistant bacteria were generally present at low abundance, with the exception of *Acinetobacter*, which was detected at relatively high levels in a specific epidermal mucus sample (Figure 6). Notably, the detection of resistant bacteria restricted to specific field samples resembled the laboratory findings, where, under relatively stable conditions such as the laboratory aquarium or dry season, potential resistant bacteria became more abundant in association with specific environmental compartments and community compositions (Figure 2A).

In samples outside the main group, the relative abundances of potential tet‐resistant bacteria were generally lower. Nevertheless, several RPP‐associated taxa, including *B. caecimuris* and *S. ruminicola*, as well as the fish intestinal bacterium *P. shigelloides* , were detected at relatively higher levels in multiple gut content samples collected during the wet season (Figure 6). This suggests that hydrological changes during the wet season altered the community membership of the microbiota, leading to shifts in the abundance and types of potential resistant bacteria.

**FIGURE 7:**
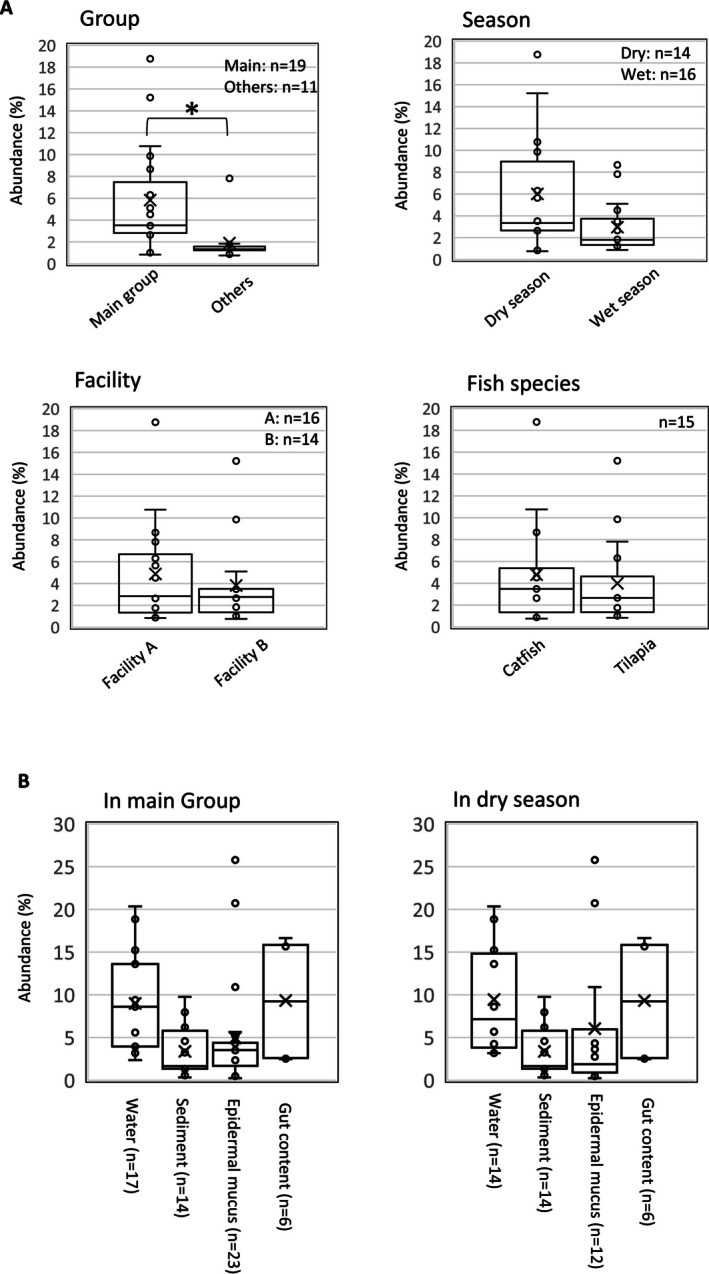
Comparison of potential tet‐resistant bacteria proportions in the bacterial flora of Indonesian aquaculture environments. (A) Based on the proportions of potential tet‐resistant bacteria detected in Figure 6, the total proportion of these bacteria was calculated for each sample and compared among bacterial flora groups (Figure 5), dry‐ and wet‐season samples, samples from Facilities (A) and (B), and samples from catfish and tilapia. The total proportions in each sample are shown as box plots. *p* < 0.01, Mann–Whitney U test. (B) Further comparisons were performed for the main group and for dry‐season samples, which tended to show higher proportions of potential tet‐resistant bacteria in panel A. Within each category, the total proportions were compared among sampling sites (gut content, epidermal mucus, water and sediment). Individual sample data from each site (Supporting Information: Sheet 2) were analysed in the same manner as in Figure 6.

We then compared the detection frequencies and relative abundances of potential tet‐resistant bacteria across multiple categories: main group versus nonmain group, dry versus wet season, Facility A versus Facility B and catfish versus tilapia (Figure 7A). Potential tet‐resistant bacteria were significantly more abundant in the main group than in other groups, with median relative abundances of 3.52% and 1.34%, respectively. Dry‐season samples showed higher median values than wet‐season samples (3.34% vs. 1.80%) (Figure 7A). Only small differences were observed between Facility A and Facility B samples (median 2.84% vs. 2.77%) and between tilapia and catfish samples (median 3.48% vs. 2.66%) (Figure 7A). Within the main group, gut content and water showed higher median relative abundances of potential tet‐resistant bacteria (9.23% and 8.60%, respectively) than mucus (3.54%) or sediment (1.65%) (Figure 7B). A similar pattern was observed within dry‐season samples, where gut content and water again showed higher median values (9.23% and 7.14%, respectively) than mucus (1.88%) or sediment (1.65%) (Figure 7B). These data indicate that samples classified within the main group, mainly from the dry season, showed increased proportions of specific potential tetracycline‐resistant bacteria, particularly in gut contents and rearing water.

## 4 Discussion

This study investigated the emergence and distribution of AMR bacteria in Indonesian aquaculture by combining controlled laboratory experiments with field surveys. We first tested how OTC exposure reshapes microbial communities under controlled conditions and then applied these insights to field surveys, thereby linking experimental mechanisms with ecological patterns. This integrative approach demonstrated that changes in microbial communities, driven by antibiotic use and hydrological conditions including seasonal variation, influence the emergence and persistence of ARB in aquaculture environments.

In the laboratory, antibiotic treatment most strongly affected the gut microbial community, suggesting that the fish gut is a key compartment in which antibiotic‐driven shifts may favour the emergence of ARB. Culture‐based isolation and time‐series read mapping further indicated that resistant strains and their closely related taxa became enriched within specific environmental compartments, implying that each compartment may serve as a reservoir for particular ARB (Figure 2). Although such time‐course monitoring is difficult to achieve in field surveys, our laboratory results demonstrate its value for detecting resistant populations and linking their occurrence to changes in community structure. Resistant strains were also recovered from control fish not exposed to antibiotics, suggesting that such bacteria may already occur naturally in the environment or enter aquarium systems through routes other than direct antibiotic treatment. This observation highlights the importance of monitoring AMR even in facilities where antimicrobials are not applied.

Field surveys of gut, mucus, water and sediment samples collected during dry and wet seasons yielded multiple tetracycline‐resistant bacteria, including *A. baumannii* , *S. flexneri* , *K. pneumoniae* , *S. marcescens* , *S. maltophilia* and *C. testosteroni* (Table 2). The detection of such bacteria, many of which are recognised as clinically important AMR‐related species, highlights a potential public health concern (Smith and Gradon 2013; Howard et al. 2012; Zivkovic Zaric et al. 2023; Paczosa Michelle and Mecsas 2016; Brooke Joanna 2012; Ahmed et al. 2013). In particular, the presence of *Comamonas* spp., which have been implicated in human infections following tropical fish exposure (Smith and Gradon 2013), illustrates the potential risk of transmission between aquaculture systems and humans. The persistence of such human‐associated ARB in aquaculture environments may involve multiple processes. Antibiotic use can disrupt native microbial communities and create ecological niches for resistant strains (He et al. 2022; Streb et al. 2025), while contamination from domestic wastewater may introduce additional human‐associated taxa (Klase et al. 2019).

Gene‐based analyses of environmental DNA suggested higher detection frequencies of tet‐resistance genes in the dry season and in certain environmental compartments. However, because nonspecific amplification was frequently observed, likely due to degraded or fragmented DNA, these seasonal and compartment‐specific patterns should be interpreted with caution. Sequencing of tet‐resistance gene amplicons nevertheless supported the presence of tetG‐associated bacteria across environmental compartments, whereas taxa associated with RPP genes were detected more specifically in gut contents. Many of these RPP‐associated taxa were obligate anaerobes characteristic of gut microbiota (Hayashi et al. 2007; Plomp and Harmsen 2024; Ge et al. 2021; Qiao et al. 2022), indicating that RPP‐type resistance is more strongly linked to intestinal environments than to external compartments.

Notably, many cultured tetracycline‐resistant isolates could not be directly linked to the detected *tetG* or RPP sequences. According to CARD database annotations, some isolates may harbour other resistance genes such as *tetA*, *tetB*, *tetC* or *tetD*. These genes were not amplified from environmental DNA (Figure S2), likely due to their very low abundance or technical limitations of PCR. Except for *Comamonas*, *Plesiomonas* and *Curvibacter* (Figure 6), most corresponding taxa were detected only at low levels or in limited samples, suggesting that these genes might exist below detection thresholds or within uncaptured subpopulations. Furthermore, BLASTn searches using *tetG* consensus sequences without restriction to type material revealed *tetG*‐like sequences in the genomes of *Pseudomonas alloputida*, *K. pneumoniae* , *A. baumannii* and *M. morganii* , indicating that these isolates may also carry *tetG* or homologous genes.

Seasonal variation strongly shaped bacterial community composition in the Indonesian aquaculture environment. In the dry season, bacterial communities were more uniform across environmental compartments and formed a dominant community configuration (Figure 5B), suggesting that relatively stable hydrological conditions promoted homogenisation of microbial communities. This dominant group also contained higher abundances of potential tet‐resistant taxa, including *C. resistens*, *C. testosteroni* , *C. lanceolatus* , *G. aquarius* and *P. lemoignei* , implying that such stabilised community structures may favour the accumulation of specific resistant bacteria. A similar tendency was observed in the laboratory aquarium system, where bacterial communities across compartments became more similar over time, supporting the idea that limited water exchange can increase community homogenisation and facilitate enrichment of particular resistant taxa. By contrast, wet‐season samples were more heterogeneous and showed reduced detection of the taxa dominant in the dry season, while additional RPP‐associated groups appeared, particularly in gut samples. These patterns suggest that increased water exchange during the wet season altered community membership and thereby changed the abundance and composition of potential resistant bacteria outside the dominant dry‐season group. Such shifts may reflect both the dispersal of ARB through water and the introduction of additional bacteria from external sources. Changes in resistant bacteria detected in gut samples during the wet season may also have been indirectly driven by hydrological effects on water and sediment microbial communities, which in turn influenced the gut microbiota (He et al. 2023; Yang et al. 2022).

Quantitative analysis indicated that gut content and water were the compartments in which potential tet‐resistant bacteria were most frequently detected, particularly in main‐group samples from the dry season (Figure 7B). This pattern is consistent with the laboratory results showing that antibiotic exposure primarily affected the gut microbiota. The detection of gut‐associated bacteria in water samples further supports the idea that the fish gut functions as a primary site for the emergence of ARB, from which resistant bacteria may be released into the surrounding water through faecal excretion and subsequently accumulate under dry‐season conditions.

## 5 Conclusion

Antibiotic administration primarily affected the fish gut microbiota, supporting the gut as a primary site for the emergence of resistant bacteria. At the same time, distinct environmental compartments may serve as reservoirs for specific ARB populations, while surrounding water appears to function as an important medium for their accumulation and dispersal. Seasonal variation, particularly hydrological change, further influenced the composition and abundance of these resistant communities.

## Author Contributions

**Hajime Nakatani:** conceptualisation, investigation, methodology, project administration, supervision, visualisation, data curation, funding acquisition, writing – original draft, writing – review and editing. **Noor Hidhayati:** investigation. **Dien Arista Anggorowati:** investigation, methodology, writing – review and editing. **Aoi Kaji:** investigation. **Kaho Tobioka:** investigation. **Saki Ishiguro:** investigation. **Stephanie Angela Yosiano:** investigation. **Marsiti Apriastini:** investigation. **Khairul Anam:** investigation, methodology, writing – review and editing. **Dwi Susilaningsih:** investigation, methodology, writing – review and editing. **Koji Mitsui:** investigation. **Kotone Yamamoto:** investigation. **Sae Tanaka:** investigation. **Idham Sumarto Pratama:** investigation. **Asep Ridwanudin:** investigation. **Tomoko Arakawa:** conceptualisation, methodology, funding acquisition. **Fumiyoshi Okazaki:** conceptualisation, investigation, methodology, project administration, supervision, funding acquisition, writing – review and editing.

## Funding

This work was supported by the Sumitomo Foundation.

## Ethics Statement

In the country where this study was conducted, ethical approval is not currently required for experiments involving fish, and no institutional review board system exists for fish research. Nevertheless, all experimental procedures involving fish were carried out in accordance with the *Guidelines for the Use of Fishes in Research* (American Fisheries Society, 2014), with careful attention to humane treatment and minimisation of animal distress.

## Conflicts of Interest

The authors declare no conflicts of interest.

## Supporting information


**Figure S1:** Administration of OTC in aquarium environment and isolation of OTC‐resistant bacteria.
**Figure S2:** Detection of tet efflux pump genes from environmental DNA in Indonesian aquaculture by PCR.
**Figure S3:** Detection of RPP genes from environmental DNA in Indonesian aquaculture by PCR.
**Figure S4:** Bacterial flora analysis of samples collected from Indonesian aquaculture environments.
**Table S1:** Primers used in this study.
**Table S2:** OTUs increased or decreased in abundance by OTC administration.
**Table S3:** Isolated bacteria with tetracycline resistance from tilapia and catfish pond.
**Data Sheets:**Supplemental 1:
**Data Sheet:** 1. Operational taxonomic units (OTUs) identified in the samples collected from zebrafish aquaria in laboratory experiments. 2. Rarefaction analysis for phylogenetic diversity (PD) in laboratory experiments. 3. 16S rRNA gene sequences of bacterial isolates cultured in laboratory experiments after OTC administration. 4. Differential abundance analysis of OTUs in OTC‐treated versus untreated groups from week 4 samples.
**Data Sheets:**Supplemental 2:
**Data Sheet:** 5. Operational taxonomic units (OTUs) identified in the samples collected from Indonesian aquaculture facilities. 6. Consensus sequences of tetracycline resistance genes identified from the samples collected from Indonesian aquaculture facilities. 7. Potential tetracycline‐resistant host bacterial strains. 8. Full‐length 16S rRNA gene sequences of the bacterial strains that highly similar to those of isolated tet‐resistant bacteria from Indonesian aquaculture samples. 9. Reference 16S rRNA gene sequences of potential host bacteria for tet‐resistance genes. 10. Rarefaction analysis for phylogenetic diversity (PD) in the sample from Indonesian aquaculture facilities.

## Data Availability

The data that support the findings of this study are openly available in DDBJ Sequence Read Archive at https://www.ddbj.nig.ac.jp/dra/index.html, reference number DRR659587‐DRR659632,DRR686378‐DRR686407,DRR686408‐DRR686412.
