# Supplementary material for: Microbiota Composition Shaped by Seasonal Variation and Environmental Compartments Affects the Abundance of Antimicrobial‐Resistant Bacteria in Indonesian Aquaculture
Source: Environ Microbiol Rep. 2026 Apr 14;18(2):e70337. doi: 10.1111/1758-2229.70337 (PMC13078929; doi:10.1111/1758-2229.70337)
Supplement: Supplementary file 1 — Figure S1: Administration of OTC in aquarium environment and isolation of OTC‐resistant bacteria. Figure S2: Detection of tet efflux pump genes from environmental DNA in Indonesian aquaculture by PCR. Figure S3: Detection of RPP genes from environmental DNA in Indonesian aquaculture by PCR. Figure S4: Bacterial flora analysis of samples collected from Indonesian aquaculture environments. Table S1: Primers used in this study. Table S2: OTUs increased or decreased in abundance by OTC administration. Table S3: Isolated bacteria with tetracycline resistance from tilapia and catfish pond. Data Sheets:Supplemental 1: Data Sheet: 1. Operational taxonomic units (OTUs) identified in the samples collected from zebrafish aquaria in laboratory experiments. 2. Rarefaction analysis for phylogenetic diversity (PD) in laboratory experiments. 3. 16S rRNA gene sequences of bacterial isolates cultured in laboratory experiments after OTC administration. 4. Differential abundance analysis of OTUs in OTC‐treated versus untreated groups from week 4 samples. Data Sheets:Supplemental 2: Data Sheet: 5. Operational taxonomic units (OTUs) identified in the samples collected from Indonesian aquaculture facilities. 6. Consensus sequences of tetracycline resistance genes identified from the samples collected from Indonesian aquaculture facilities. 7. Potential tetracycline‐resistant host bacterial strains. 8. Full‐length 16S rRNA gene sequences of the bacterial strains that highly similar to those of isolated tet‐resistant bacteria from Indonesian aquaculture samples. 9. Reference 16S rRNA gene sequences of potential host bacteria for tet‐resistance genes. 10. Rarefaction analysis for phylogenetic diversity (PD) in the sample from Indonesian aquaculture facilities. [file EMI4-18-e70337-s001.docx]

**Supplemental materials**

**Microbiota Composition Shaped by Seasonal Variation and Environmental Compartments Affects the Abundance of Antimicrobial-Resistant Bacteria in Indonesian Aquaculture**

Hajime Nakatani^1*^, Noor Hidhayati^2^, Dien Arista Anggorowati^3^, Aoi Kaji^4^, Kaho Tobioka^4^, Saki Ishiguro^4^, Stephanie Angela Yosiano^2^, Marsiti Apriastini^2^, Khairul Anam^2^, Dwi Susilaningsih^2^, Koji Mitsui^4^, Kotone Yamamoto^4^, Sae Tanaka^4^, Idham Sumarto Pratama^3^, Asep Ridwanudin^5^, Tomoko Arakawa^6^ and Fumiyoshi Okazaki^4*^

^1^Department of Biomolecular Engineering, Graduate School of Engineering, Nagoya University, Furo-cho, Chikusa, Nagoya, Aichi, Japan

^2^Research Center for Applied Microbiology, Research Organization for Life Sciences and Environment, National Research and Innovation Agency (BRIN), Jl. Raya Jakarta-Bogor Km 46, Cibinong, 16911, Indonesia.

^3^Research Center for Marine and Land Bioindustry, National Research and Innovation Agency (BRIN), Jl. Raya Senggigi, Kodek Bay, Pemenang, Nort Lombok, West Nusa Tenggara 83352, Indonesia.

^4^Department of Life Sciences, Graduate School of Bioresources, Mie University, 1577 Kurimamachiya, Tsu, Mie 514-8507, Japan

^5^Research Center for Applied Zoology, National Research and Innovation Agency (BRIN), Jl. Raya Jakarta-Bogor Km 46, Cibinong, 16911, Indonesia

^6^LaLa Product Ltd. Tsurumai, Showa-ku, Nagoya, Aichi, Japan

| Table S1. Primers used in this study | | |  |  |
| --- | --- | --- | --- | --- |
| **Target** | **Name** | **Sequence (5'-3')** | | **Reference** |
| 16S rRNA gene full | 9F | GAGTTTGATCCTGGCTCAG | | - |
|  | 1510R | GGCTACCTTGTTACGA | |  |
| 16S rRNA gene V4 | NGS v4F | TCGTCGGCAGCGTCAGATGTGTATAAGAGACAGGTGYCAGCMGCCGCGGTAATWC | | Earth Microbiome Project ^1)^ |
|  | NGS v4R | GTCTCGTGGCTCCGGAGATGTGTATAAGAGACAGGGACTACHVGGGTWTCTAAT | |  |
| 16S rRNA gene V1-V2 | NGS63F | TCGTCGGCAGCGTCAGATGTGTATAAGAGACAGCAGGCCTAACACATGCAAGTC | | Nakatani H. et al.^2)^ |
|  | NGS338R | GTCTCGTGGGCTCGGAGATGTGTATAAGAGACAGGCTGCCTCCCGTAGGAGT | |  |
| RPP ecept for o*trA* | Ribo2-FWa | GGMCAYRTGGATTTYWTIGC | | Mackie R. I. et al.^3)^ |
|  | Ribo2-RV | TCIGMIGGIGTRCTIRCIGGRC | |  |
| *tetBP* | TetB/P-FW | AAAACTTATTATATTATAGTG | |  |
|  | TetB/P-RV | TGGAGTATCAATAATATTCAC | |  |
| *tetM* | TetM-FW | ACAGAAAGCTTATTATATAAC | |  |
|  | TetM-RV | TGGCGTGTCTATGATGTTCAC | |  |
| *tetO* | TetO-FW | ACGGARAGTTTATTGTATACC | |  |
|  | TetO-RV | TGGCGTATCTATAATGTTGAC | |  |
| *tetQ* | TetQ-FW | AGAATCTGCTGTTTGCCAGTG | |  |
|  | TetQ-RV | CGGAGTGTCAATGATATTGCA | |  |
| *tetS* | TetS-FW | GAAAGCTTACTATACAGTAGC | |  |
|  | TetS-RV | AGGAGTATCTACAATATTTAC | |  |
| *tetT* | TetT-FW | AAGGTTTATTATATAAAAGTG | |  |
|  | TetT-RV | AGGTGTATCTATGATATTTAC | |  |
| *tetW* | TetW-FW | GAGAGCCTGCTATATGCCAGC | |  |
|  | TetW-RV | GGGCGTATCCACAATGTTAAC | |  |
| *otrA* | OTR-FW | GGCATYCTGGCCCACGT | |  |
|  | OTR-RV | CCCGGGGTGTCGTASAGG | |  |
| *tetA, tetC* | tetAC-F | CGCYTATATYGCCGAYATCAC | | Furushita M. et al.^4)^ |
|  | tetAC-R | CCRAAWKCGGCWAGCGA | |  |
| *tetB, tetD* | tetBDEFHJ-F | GGDATTGGBCTTATYATGCC | |  |
|  | tetBD-R | ATMACKCCCTGYAATGCA | |  |
| *tetG, tetY* | tetGY-F | TATGCRTTKATGCAGGTC | |  |
|  | tetGY-R | GACRAKCCAAACCCAACC | |  |
| *tetE, tetH, tetJ* | tetBDEFHJ-F | - | |  |
|  | tetEHJ-R | AWDGTGGCDGGAATTTG | |  |
| *tetL* | tetLF | TCGTTAGCGTGCTGTCATTC | |  |
|  | tetLR | GTATCCCACCAATGTAGCCG | |  |
| *tetK* | tetKF | TCGATAGGAACAGCAGTA | | Ng LK. et al.^5)^ |
|  | tetKR | CAGCAGATCCTACTCCTT | |  |

1. <http://www.earthmicrobiome.org/emp-standard-protocols/16s/>
2. Nakatani H. et al. (2022) *Biology* 11:1249
3. Mackie R. I. et al. (2001) *Appl Environ Microbiol.* 67(1):22-32.
4. Furushita M. et al. (2003) *Appl Environ Microbiol.* 69:5336-5342.
5. Ng LK. et al. (2001) *Mol Cell Probes.*15:209-215.


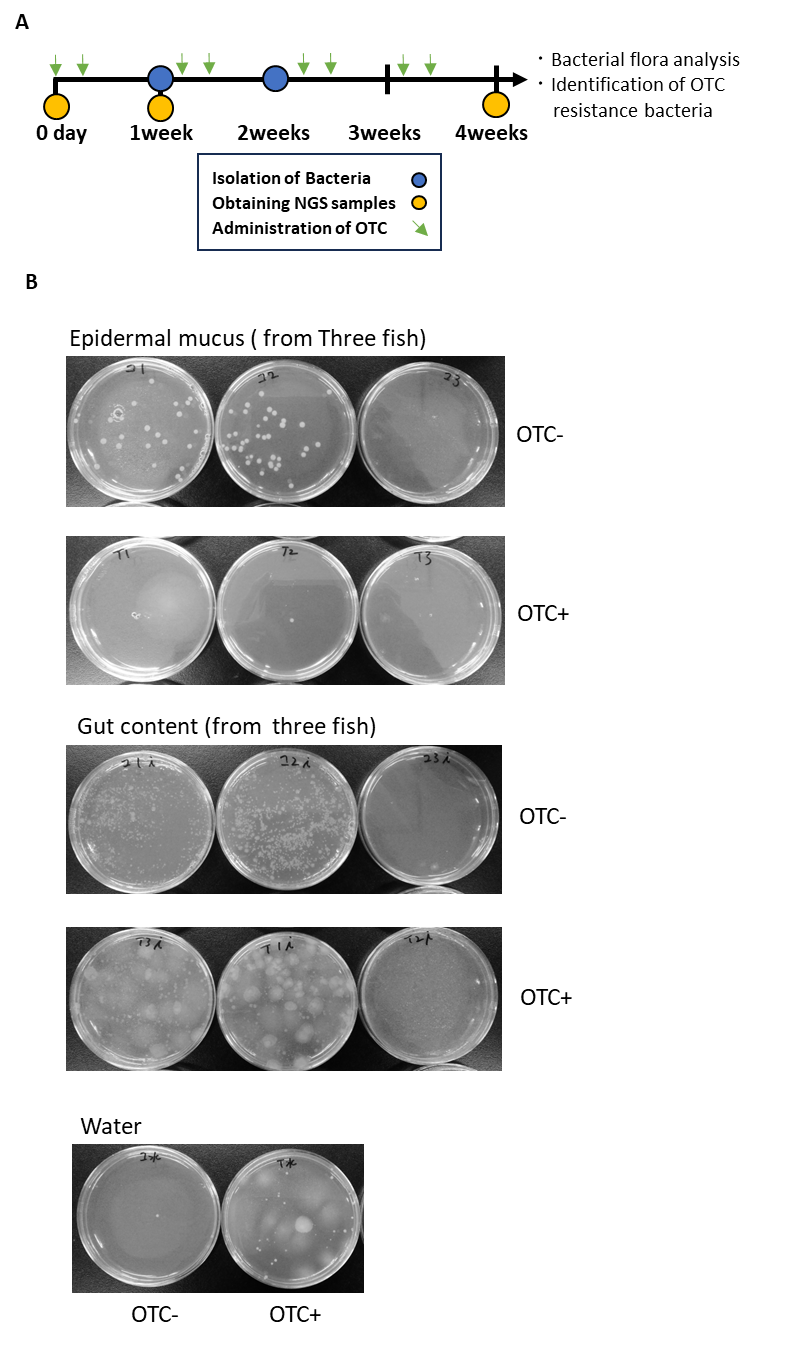


**Fig. S1. Administration of OTC in aquarium environment and isolation of OTC-resistant bacteria.** (A) OTC administration schedule and sampling for zebrafish reared in a recirculating tank. The experiment lasted for four weeks, with separate tanks for OTC-treated and non-treated groups. In the OTC-treated group, 5 mg of OTC was mixed with feed and administered twice a week. Samples of fish epidermis, gut contents, and water were collected before the experiment, at the first week, and at the fourth week for bacterial flora analysis. Additionally, attempts to isolate OTC-resistant bacteria from the epidermis, gut contents, and water were made during the first and second weeks. (B) Examples of OTC-resistant bacterial colonies isolated on R2A agar medium during the first week. OTC-resistant bacteria were isolated from both the OTC-treated and non-treated groups.

Table S2. OTUs increased or decreased in abundance by OTC administration

Increased

| No. | Sub  group | Hold change | FDR p-value | Top-hit Taxon | Identity(%) |
| --- | --- | --- | --- | --- | --- |
| 1 | 4w_others | 138.9 | 0.034 | n/a (*Phycisphaeraceae*) | - |
| 2 | 4w_gut | 451.1 | 0.00006 | *Luteolibacter flavescens* strain GKX | 97.4 |
| 3 | 4w_gut | 3343.7 | 0.011 | *Fimbriiglobus ruber* strain SP5 | 94.8 |
| 4 | 4w_gut | 186.8 | 0.011 | *Pseudonocardia tritici* strain NEAU-YY211 | 100 |
| 5 | 4w_gut | 119.2 | 0.011 | *Reyranella aquatilis* strain Seoho-37 | 100 |
| 6 | 4w_gut | 181.8 | 0.013 | *Rhabdothermincola salaria* strain EGI L10124 | 96.7 |
| 7 | 4w_gut | 103.8 | 0.015 | n/a (*Pirellulaceae*) | - |
| 8 | 4w_gut | 90.2 | 0.015 | *Clostridium swellfunianum* strain S11-3-10 | 98.7 |
| 9 | 4w_gut | 79.3 | 0.015 | *Trinickia diaoshuihuensis* strain NEAU-SY24 | 94.8 |
| 10 | 4w_gut | 1349.2 | 0.021 | *Luteolibacter flavescens* strain GKX | 95.4 |
| 11 | 4w_gut | 1279.0 | 0.022 | n/a (*Phycisphaerales*) | - |
| 12 | 4w_gut | 71.7 | 0.024 | *Nordella oligomobilis* strain N21 | 99.3 |
| 13 | 4w_gut | 51.7 | 0.024 | *Reyranella aquatilis* strain Seoho-37 | 94.1 |
| 14 | 4w_gut | 1229.0 | 0.036 | n/a (*Pirellulales*) | - |
| 15 | 4w_gut | 72.6 | 0.036 | *Clostridium punense* strain BLPYG-8 | 98.7 |
| 16 | 4w_gut | 49.4 | 0.064 | *Shewanella putrefaciens* strain ATCC 8071 | 98.7 |
| 17 | 4w_gut | 39.2 | 0.071 | *Bosea minatitlanensis* strain AMX51 | 100 |
| 18 | 4w_gut | 40.4 | 0.072 | *Bacillus tropicus* strain MCCC 1A01406 | 100 |
| 19 | 4w_gut | 41.7 | 0.072 | n/a | - |
| 20 | 4w_gut | 34.2 | 0.084 | n/a (*Chloroflexi*) | - |
| 21 | 4w_gut | 35.5 | 0.094 | n/a (*Bacilli)* | - |

decreased

| No. | Sub  group | Hold change | FDR p-value | Top-hit Taxon | Identity(%) |
| --- | --- | --- | --- | --- | --- |
| 1 | 4w_others | -228.3 | 0.013 | *Shewanella* | - |
| 2 | 4w_gut | -12093.3 | 0.00016 | *Pseudomonas otitidis* strain MCC10330 | 100 |
| 3 | 4w_gut | -153.7 | 0.021 | n/a (*Malacoplasma*) | - |
| 4 | 4w_gut | -112.6 | 0.05 | n/a (*Malacoplasma*) | - |
| 5 | 4w_gut | -99.4 | 0.06 | n/a (*Malacoplasma*) | - |
| 6 | 4w_gut | -68.4 | 0.07 | n/a (*Malacoplasma*) | - |

Table S3. Isolated bacteria with tetracycline resistance from tilapia and catfish pond

| **Facility** | **Sample** | **Top-hit taxon** | **Top-hit strains** | **Similarity (%)** | **Completeness (%)** |
| --- | --- | --- | --- | --- | --- |
| A | Sediment | *ー* |  |  |  |
| A | Sediment | *ー* |  |  |  |
| A | Sediment | *ー* |  |  |  |
| A | Sediment | *Shigella flexneri* | ATCC 29903 | 100.00 | 50.1 |
| A | Sediment | *Shigella flexneri* | ATCC 29903 | 99.56 | 46.5 |
| A | Sediment | *Acinetobacter haemolyticus* | CIP 64.3 | 98.77 | 50.1 |
| A | Sediment | *Acinetobacter baumannii* | ATCC 19606 | 98.31 | 52.7 |
| A | Water | *Curvibacter lanceolatus* | ATCC 14669 | 98.03 | 17.5 |
| A | Water | *Rivihabitans pingtungensis* | Npb-03 | 99.39 | 56.0 |
| A | Water | *Pseudomonas alloputida* | Kh7 | 100.00 | 52.5 |
| A | Water | *ー* |  |  |  |
| A | Water | *ー* |  |  |  |
| A | Water | *ー* |  |  |  |
| A | Water | *ー* |  |  |  |
| A | Water | *ー* |  |  |  |
| A | Water | *Vogesella urethralis* | YM-1 | 99.47 | 52.4 |
| A | Water | *ー* |  |  |  |
| A | Mucus | *Chryseobacterium lecithinasegens* | PAGU 2197 | 99.22 | 53.2 |
| A | Mucus | *Serratia marcescens* | ATCC 13880 | 99.29 | 48.0 |
| A | Mucus | *Serratia marcescens* | ATCC 13880 | 98.99 | 13.6 |
| A | Mucus | *Serratia marcescens* | ATCC 13880 | 98.63 | 55.0 |
| A | Sediment | *Serratia marcescens* | ATCC 13880 | 97.35 | 38.9 |
| A | Mucus | *Serratia marcescens* | ATCC 13880 | 100.00 | 46.1 |
| A | Mucus | *Serratia marcescens* | ATCC 13880 | 99.85 | 45.7 |
| A | Mucus | *Vogesella urethralis* | YM-1 | 99.49 | 53.9 |
| A | Mucus | *Comamonas testosterone* | ATCC 11996 | 100.00 | 55.6 |
| B | Water | *Shigella flexneri* | ATCC 29903 | 100.00 | 49.9 |
| B | Water | *Shigella flexneri* | ATCC 29903 | 100.00 | 50.1 |
| B | Sediment | *ー* |  |  |  |
| B | Sediment | *ー* |  |  |  |
| B | Sediment | *Plesiomonas shigelloides* | NCTC 10360 | 99.41 | 34.9 |
| B | Sediment | *ー* |  |  |  |
| A | Water | *Massilia aerilata* | 5516S-11 | 99.66 | 41.0 |
| A | Water | *Chryseobacterium cucumeris* | GSE06 | 99.87 | 52.5 |
| A | Water | *Citrobacter freundii* | DSM 30039 | 99.87 | 51.2 |
| A | Water | *Providencia alcalifaciens* | DSM 30120 | 99.87 | 51.0 |
| A | Water | *ー* |  |  |  |
| A | Water | *Klebsiella pneumoniae subsp. Ozaenae* | ATCC 11296 | 99.61 | 52.9 |
| A | Water | *ー* |  |  |  |
| A | Water | *Providencia alcalifaciens* | DSM 30120 | 99.72 | 49.1 |
| A | Sediment | *Acinetobacter modestus* | NIPH 236 | 99.61 | 52.6 |
| A | Sediment | *Vogesella urethralis* | YM-1 | 99.47 | 51.9 |
| A | Sediment | *ー* |  |  |  |
| A | Sediment | *Shigella flexneri* | ATCC 29903 | 99.73 | 50.9 |
| A | Sediment | *Morganella morganii subsp. Sibonii* | DSM 14850 | 99.33 | 51.1 |
| A | Sediment | *Morganella morganii subsp. Sibonii* | DSM 14850 | 99.06 | 51.1 |
| A | Sediment | *ー* |  |  |  |
| A | Sediment | *Acinetobacter gyllenbergii* | CIP 110306 | 100.00 | 33.6 |
| A | Intestine | *Morganella morganii subsp. Morganii* | ATCC 25830 | 99.87 | 52.3 |
| A | Intestine | *Morganella morganii subsp. Morganii* | ATCC 25830 | 99.86 | 47.2 |
| A | Intestine | *Morganella morganii subsp. Morganii* | ATCC 25830 | 98.89 | 49.3 |
| A | Intestine | *Morganella morganii subsp. Morganii* | ATCC 25830 | 99.87 | 51.2 |
| A | Intestine | *Morganella morganii subsp. Sibonii* | DSM 14850 | 99.06 | 51.1 |
| A | Intestine | *Morganella morganii subsp. Sibonii* | DSM 14850 | 99.22 | 54.4 |
| A | Intestine | *Morganella morganii subsp. Sibonii* | DSM 14850 | 99.06 | 52.6 |
| A | Intestine | *Morganella morganii subsp. Sibonii* | DSM 14850 | 99.22 | 53.9 |
| A | Water | *Morganella morganii subsp. Morganii* | ATCC 25830 | 99.09 | 52.6 |
| A | Water | *Rivihabitans pingtungensis* | Npb-03 | 99.87 | 53.5 |
| A | Water | *ー* |  |  |  |
| A | Water | *Rivihabitans pingtungensis* | Npb-03 | 99.51 | 55.6 |
| A | Mucus | *Chryseobacterium ureilyticum* | DSM 18017 | 99.18 | 34.1 |
| A | Mucus | *ー* |  |  |  |
| A | Mucus | *Undibacterium squillarum* | CMJ-15 | 99.83 | 40.5 |
| A | Mucus | *Acinetobacter tibetensis* | Y-23 | 99.03 | 53.0 |
| B | Mucus | *Stenotrophomonas maltophilia* | MTCC 434 | 98.94 | 45.3 |
| B | Mucus | *Chryseobacterium hispalense* | DSM 25574 | 98.71 | 48.8 |
| B | Mucus | *ー* |  |  |  |
| B | Mucus | *Paucimonas lemoignei* | LMG 2207 | 94.49 | 33.7 |
| B | Mucus | *Pseudoduganella violacea* | CAVIO | 98.89 | 56.0 |

Yellow cells: Isolates from tilapia pond. Grey cells: Isolates from catfish pond.


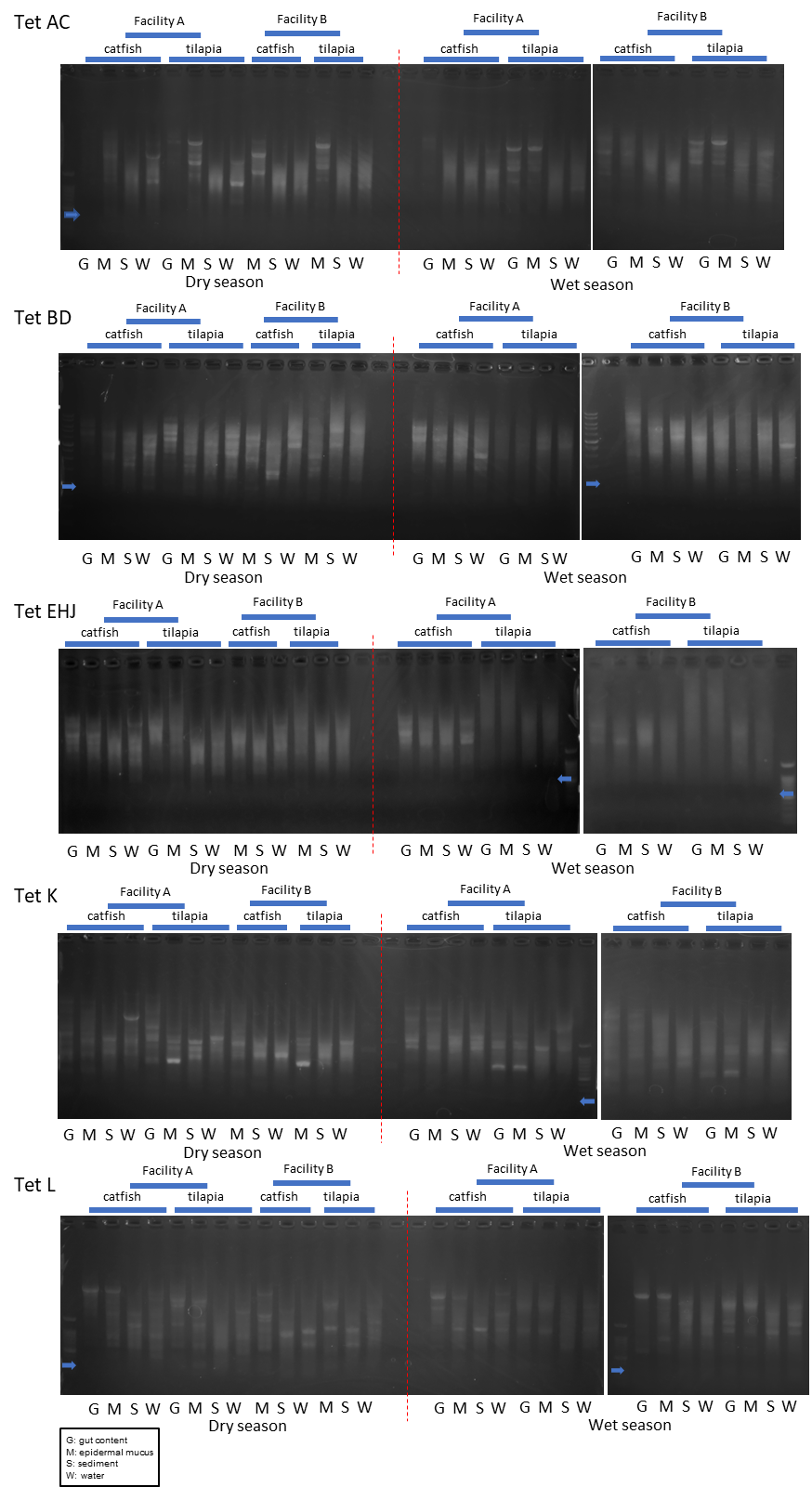


**Fig. S2. Detection of tet efflux pump genes from environmental DNA in Indonesian aquaculture by PCR.** Detection of efflux pump genes using universal primers for *tetA/C*, *tetB/D*, *tetE/H/J,* *tetK*, *tetL* via PCR. The expected amplicon size was shown by arrows.


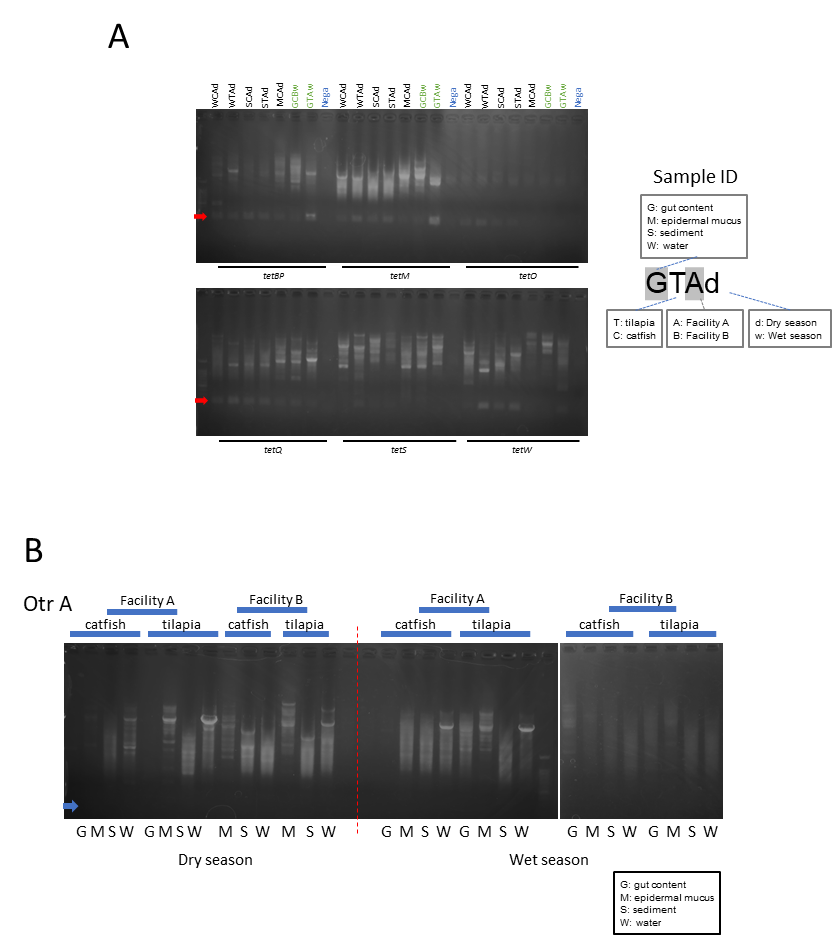


**Fig. S3. Detection of RPP genes from environmental DNA in Indonesian aquaculture by PCR.** Detection of RPP genes using specific primers for *tetBP*, *tetM*, *tetO*, *tetQ*, *tetS*, *tetW*, and *otrA* via PCR. The expected amplicon size was shown by arrows.


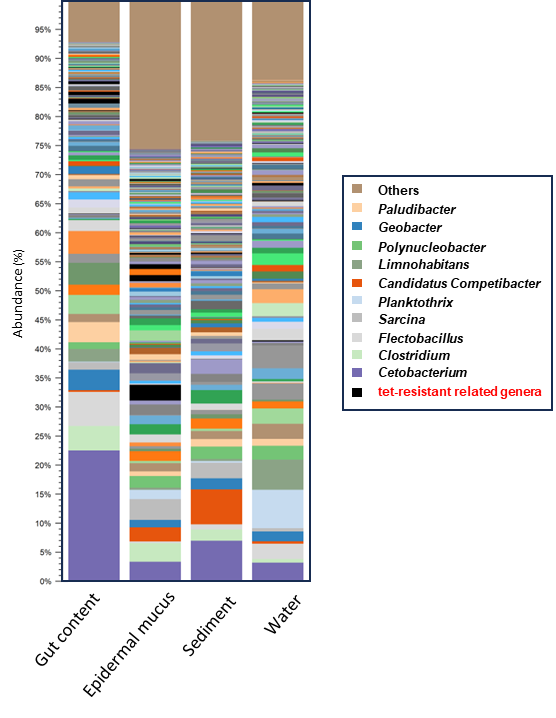


**Fig. S4. Bacterial Flora Analysis of Samples Collected from Indonesian Aquaculture Environments.** The bacterial flora of different sampling sites in aquaculture environments was analyzed, and the proportion of bacterial genera in each sampling part was displayed as a stacked bar chart. The data represent the combined analysis results of multiple samples collected from each part. Bacterial genera associated with tet-resistant bacteria identified in culture experiments are indicated in black color.
